# Genome-wide methylome-based molecular pathologies associated with depression and suicide

**DOI:** 10.1038/s41386-024-02040-9

**Published:** 2024-12-07

**Authors:** Yogesh Dwivedi, Bhaskar Roy, Praveen Kumar Korla

**Affiliations:** Department of Psychiatry and Behavioral Neurobiology, University of Alabama at Birmingham, Birmingham, AL 35294 USA

**Keywords:** DNA methylation, Depression

## Abstract

Major depressive disorder (MDD) is a debilitating disorder. Suicide attempts are 5-times higher in MDD patients than in the general population. Interestingly, not all MDD patients develop suicidal thoughts or complete suicide. Thus, it is important to study the risk factors that can distinguish suicidality among MDD patients. The present study examined if DNA methylation changes can distinguish suicidal behavior among depressed subjects. Genome-wide DNA methylation was examined in the dorsolateral prefrontal cortex of depressed suicide (MDD+S; *n* = 15), depressed non-suicide (MDD−S; *n* = 17), and nonpsychiatric control (C; *n* = 16) subjects using 850 K Infinium Methylation EPIC BeadChip. The significantly differentially methylated genes were used to determine the functional enrichment of genes for ontological clustering and pathway analysis. Based on the number of CpG content and their relative distribution from specific landmark regions of genes, 32,958 methylation sites were identified across 12,574 genes in C vs. MDD+/−S subjects, 30,852 methylation sites across 12,019 genes in C vs. MDD−S, 41,648 methylation sites across 13,941 genes in C vs. MDD+S, and 49,848 methylation sites across 15,015 genes in MDD−S vs. MDD+S groups. A comparison of methylation sites showed 33,129 unique methylation sites and 5451 genes in the MDD−S group compared to the MDD+S group. Functional analysis suggested oxytocin, GABA, VGFA, TNFA, and mTOR pathways associated with suicide in the MDD group. Altogether, our data show a distinct pattern of DNA methylation, the genomic distribution of differentially methylated sites, gene enrichment, and pathways in MDD suicide compared to non-suicide MDD subjects.

## Introduction

Major depressive disorder (MDD) is the leading cause of disability worldwide and significantly contributes to the global burden of disease [1–3]. Globally, ~3.8% of the population experiences depression, whereas, in the United States, the age-standardized prevalence of depression among adults is ~18.5% [4]. MDD is a strong predictor of suicide [5–7], and the prevalence rates of suicidal ideation and suicide attempts in MDD patients are 53 and 31%, respectively [8, 9]. Also, patients with MDD have an increased risk of complete suicide after a suicide attempt [10]. Thus, identifying risk factors that can distinguish suicidality among MDD patients is of high importance.

At present, the pathophysiological mechanisms associated with MDD and suicide risk are not clearly understood. Recently, an increased understanding of epigenetic processes has opened promising avenues in understanding the molecular basis of mental disorders, including MDD [11–15]. A few studies have examined DNA methylation changes in the brains of depressed suicide subjects or suicide subjects with various mental illnesses and have shown a unique epigenetic role in governing gene vs environmental crosstalk in these disorders. However, they do not provide evidence as to how these epigenetic changes increase the risk of suicide in MDD patients. For example, using a candidate gene approach, increased DNA methylation in the GABAA receptor gene, along with DNA methyltransferase B, has been reported in the brains of individuals who died by suicide [16]. Similarly, promoter hypermethylation in tyrosine kinase B (TrkB1) and BDNF genes has been found in the prefrontal cortex of suicide completers [17]. S-adenosylmethionine decarboxylase (AMD1) and arginase (ARG2) are the other candidate genes that show hypomethylation in Wernicke’s area of suicide completers [18]. Using a genome-wide approach, DNA methylation changes in 366 promoters in hippocampi of suicide completers have been reported [19]. In the prefrontal cortex of MDD subjects, a genome-wide study identified 224 candidate regions with DNA methylation differences >10%. These regions were enriched for neuronal growth and development genes [20]. A meta-analysis, which included DNA methylation data from the prefrontal cortex and cerebellum of suicide completers and non-psychiatric controls, showed evidence for altered DNA methylation at several gene loci in both brain regions with suicide-associated differentially methylated positions enriched with functional pathways relevant to nervous system development and long-term synaptic depression [21]. These studies indicate a strong relationship between DNA methylation and depression and suicide. So far, MDD-associated increased risk of suicide has not been studied at the epigenome-wide scale to understand the global DNA methylome changes in one of the vulnerable brain areas of MDD subjects with or without a history of suicide. We hypothesize that MDD is associated with alterations in genome-wide methylation, and methylation at specific sites may contribute to an increased risk of suicide in the MDD population.

Using 850K Infinium Methylation EPIC BeadChip, the present study examined genome-wide DNA methylation in the dorsolateral prefrontal cortex (dlPFC) of MDD subjects who died by suicide (MDD+S) or causes other than suicide (MDD−S) and compared with nonpsychiatric controls (C). We chose the dlPFC brain area as it is involved in hypothalamic-pituitary-adrenal axis regulation, executive and cognitive functions, as well as emotion regulation, and has been implicated in depression and suicidal behavior [22–26]. The hyper- and hypo-methylated sites were mapped across 22 autosomes, and significantly differentially methylated gene lists were used to determine the functional enrichment of genes for ontological clustering and pathway analysis. The chromosome-wise mapping of methylated sites based on the number of CpG content and their relative distribution from specific landmark regions of genes showed significant differences between MDD+S, MDD−S, and control groups. Functional analysis suggested oxytocin, GABA, VGFA, TNFA, and mTOR pathways associated with suicide in the MDD group. Our methylation analysis also showed a significant association between MDD and suicide with genomic regions annotated to genes involved in immune modulation, synaptic plasticity, and neuronal regulation. Collectively, our study shows distinct methylome footprints in the brains of MDD suicide subjects compared to MDD non-suicide subjects and nonpsychiatric controls.

## Materials and methods

A detailed methodology of postmortem brain procurement, diagnostic procedures, histopathological examinations, tissue dissection, DNA methylation, and analyses are provided in the Supplemental section.

### Human postmortem brain samples

Approval for the study was obtained from the Institutional Review Board of the University of Alabama at Birmingham. dlPFC samples were obtained from the Maryland Brain Collection at the Maryland Psychiatric Research Center, Baltimore, MD. The cohort consisted of 48 brain samples: 15 from the MDD+S group, 17 from the MDD−S group, and 16 from the non-psychiatric disorder group (C). Tissues were collected only after a family member gave informed consent and were screened for neuropathological features. Demographic and clinical characteristics of subjects are shown in Supplementary Table S1. The psychiatric diagnosis was determined by psychological autopsy [27] using Diagnostic Evaluation After Death (DEAD) [28] and the Structured Clinical Interview for the DSM-V (SCID) [29].

### DNA methylome assays with Illumina 850K EPIC array

Genomic DNA was extracted from dlPFC using QIAamp ® genomic DNA extraction kit (Qiagen, Germany). The concentration of extracted genomic DNA was measured with Nanodrop (Thermo Scientific, USA) and the OD260/OD280 ratio was used for detecting any residual contaminants in DNA preparation. Isolated DNA samples were treated with bisulfite reagents following the manufacturer’s protocol using a DNA methylation kit (Zymo Research, USA). Treated DNA samples after purification were then hybridized with the 850K Infinium Methylation EPIC BeadChip (Illumina Inc., USA), which covered over 850,000 methylation sites located in CpG islands, genes, transcription binding sites, open chromatin regions, and enhancers at single-nucleotide resolution. Both unmethylated and methylated CpGenome controls were included, and duplicates of a pooled DNA sample were incorporated in each array to assess inter-array consistency.

### Differential methylation data analysis and visualization

All samples passed QC procedures. Signal intensities and raw methylation β values were extracted from Illumina’s Genome-Studio software R package ChAMP (https://bioconductor.org/packages/release/bioc/html/ChAMP.html). The methylation β values were generated based on normalized signal intensities and after background subtraction using negative control probes. Differential methylation analyses were conducted across four groups (C vs. MDD+/−S, C vs. MDD−S, C vs. MDD+S, MDD−S vs. MDD+S) using a threshold of ±0.2 β value (*p* < 0.05). An outline of the methylation analysis following the genomic feature extraction steps is presented in Supplementary Fig. S1. Quantile-Quantile plots were employed to validate the distributional assumptions of methylation data across comparisons. We also applied feature extraction tools to annotate the significant methylation sites for relative chromosomal localization of methylation site, associated gene name, CpG islands information, and prediction-based functional clustering. The hyper- and hypomethylated sites were mapped across 22 autosomes using PhenoGram (http://visualization.ritchielab.org/phenograms/plot). Additionally, the Manhattan plot (https://jee-hyoung-kim-9.shinyapps.io/Manhattan_Plot/) was used to visualize the chromosome-wide distribution of DM sites. Gene ontology enrichment analyses were performed using DAVID and Metascape tools to identify enriched biological pathways and functional clusters associated with differentially methylated genes.

### Covariate analyses

Age, PMI, pH of the brain, and RIN were correlated with the top 25 methylation sites in the MDD groups using the Pearson correlation coefficient. Also, the effect of sex, race, and antidepressant toxicology, was evaluated by comparing the control group with the MDD group.

### Tissue-specific expression and regulatory element analysis

Brain-specific expression profiles of key methylated genes were examined using the Genotype-Tissue Expression (GTEx) database, confirming their tissue specificity and relevance in brain function.

### cDNA synthesis and qPCR

We used 500 ng RNA to synthesize cDNA using M-MLV Reverse Transcriptase (Invitrogen, Grand Island, NY, USA) and oligo (dT) primer as previously described [30]. Gene-specific forward and reverse primers are provided in Supplementary Table S2. Fold-change was calculated following Livak’s ΔΔCt method [31]. For the VEGFA and BRAF expression study, TaqMan probe chemistry was used (Invitrogen, USA).

## Results

### Subject comparison

As shown in Supplementary Table S1, there were no significant differences in PMI, brain pH, RIN, or age between control and MDD+/−S subjects. Also, we did not find significant differences in PMI, brain pH, RIN, or age when the control group was compared with MDD+S, MDD−S, or when the MDD+S group was compared with the MDD−S group.

### Principal component analysis (PCA) and distributional assumptions

Principal Component Analysis (PCA) was performed to explore potential clustering patterns among study groups based on sex distribution, given the unequal representation across groups. The analysis did not reveal distinct clustering patterns based on sex for the entire cohort (Supplementary Fig. S2A) or when analyzed separately for control, MDD+S, and MDD−S groups (Supplementary Fig. S2B). This indicated that sex was not a major factor of variability in methylation patterns. Furthermore, Q-Q plots were employed to validate the distributional assumptions of methylation data across comparison groups (Supplementary Fig. S3A–D). The observed versus expected plots demonstrated close adherence to expected distributions, confirming the appropriateness of parametric tests used for differential methylation analysis. These statistical validations supported the robustness of subsequent findings related to differential methylation across study groups.

### Differential methylation analysis

**Fig. 1:**
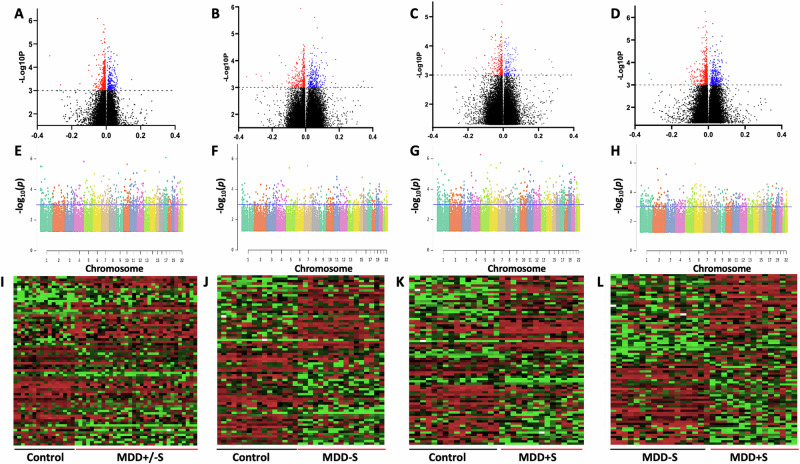
Volcano plots, Manhattan plots, and Heat maps are based on genome-wide differential methylation changes between groups. Differential methylation sites based on beta value differences are shown in volcano plots following four separate group-wise comparisons (**A**–**D**). In the plot, the red color indicates hypermethylation, the blue color indicates hypomethylation, and sites with black color are non-significant sites. Differential methylation (beta value difference) and significance level with a threshold of log10 *P* value (log10*P* > 3) are color labeled with red and blue for **A** C vs. MDD+/−S, **B** MDD−S vs. MDD+S, **C** C vs. MDD−S and **D** C vs. MDD+S comparisons. Chromosome-wise annotation of differential methylation sites are shown with Manhattan plots for four separate group-wise comparisons (**E**–**H**). Distributional differential methylation positioning between **E** C vs. MDD+/−S, **F** C vs. MDD+S, **G** C vs. MDD−S, and **H** MDD+S vs. MDD−S is presented by chromosomal location (x-axis). Each color block indicates an individual chromosome, and the methylation sites are shown in a dotted format. The blue line on the individual plot indicates the threshold of significance as determined by the Bonferroni correction method. Methylated sites with –log10 *P* value > 3 were set above the blue horizontal line. Heat maps showing the top 100 differentially methylated sites, including hyper and hypo-methylation status, across four separate group-wise analyses (**I**–**L**). Hypermethylated (beta value > 0.9) sites are shown in red, and hypomethylated (beta value < 0.004) sites are shown in green color as determined based on comparisons between **I** C vs. MDD+/−S, J) C vs. MDD−S, **K** C vs. MDD+S, and **L** MDD+S vs. MDD−S groups.

Differential methylation analyses were conducted across various groups: C vs. MDD+/−S, C vs. MDD−S, C vs. MDD+S, and MDD−S vs. MDD+S. These analyses identified numerous genomic loci exhibiting significant alterations in methylation status, providing insights into the molecular underpinnings of MDD and suicidal behavior. Detailed differential methylation analysis and genomic features across the four group comparisons are provided in Supplementary Tables S3–S6. Volcano plots (Fig. 1A–D) visually represent these differences, with hypermethylated sites marked in red and hypomethylated sites in blue, each annotated with their respective probe IDs. The plots highlighted regions across the genome where methylation changes exceeded statistical significance thresholds, offering a comprehensive view of differential methylation patterns associated with MDD and suicide. A chromosome-wise distribution of methylation sites across various comparisons is represented as Manhattan plots showing the most significant sites with -log10_*P*_ > 3 on the y-axis and ranked chromosomes on the X-axis (Fig. 1E–H). The blue line on each plot defines the significance level determined by the Bonferroni correction method.

The top 100 differentially methylated sites (50 hyper- and 50 hypomethylated sites) were used to generate heatmaps for all four comparisons (Fig. 1I–L), where methylation variations between the groups are shown with changes in color intensity based on β-value differences. These differentially methylated sites are listed in Supplementary Tables S7–S10 for each comparison. Comparison between C and total MDD subjects with and without suicide (MDD+/−S) identified overall 32,958 differentially methylated sites (*p* < 0.05) spanning across 12,574 genes. Differential methylation analysis between C and MDD−S identified differentially methylated 30,852 sites (*p* < 0.05) spanning across 12,019 genes, showing their association with the non-suicide MDD group. To determine the differential methylation profile in MDD subjects who died by suicide, a comparison was made between C and MDD+S groups, which identified 41,647 differentially methylation sites (*p* < 0.05), mapping across 13,941 genes. Lastly, a comparison between the MDD groups (MDD+S vs. MDD−S) showed 49,848 differentially methylated sites (*p* < 0.05), which were found to span across 15,015 genes.

### Unique methylation pattern in MDD with and without suicide groups and genomic features of differentially methylated sites

**Fig. 2:**
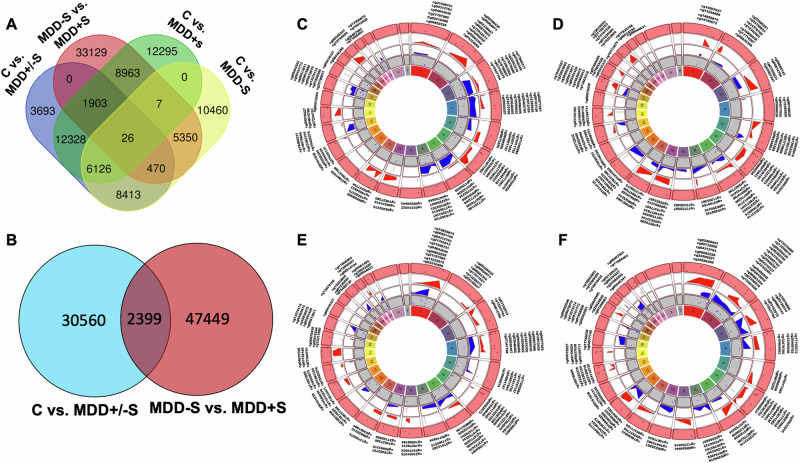
Venn diagrams and circular plots illustrating the distinct and overlapping sets of differentially methylated sites (DMs) and their chromosome-wise annotation across the genome in four sets of comparisons. In panel **A**, the focus is on highlighting the unique methylation sites identified when comparing DMs individually obtained from analyses contrasting C vs. MDD+/−S, C vs. MDD−S, C vs. MDD+S, and MDD+S vs. MDD−S. Panel **B** depicts a Venn diagram showcasing the unique and shared methylation sites resulting from the differential methylation analysis conducted between C vs. MDD+/−S and MDD+S vs. MDD−S. Circular plots are drawn to visually represent the chromosomal annotation of differential methylation sites (DMs) across the genome. In panels **C**–**F**, Circos plots depict the chromosome-wise distribution of the most significant differential methylation sites (both hyper and hypo) identified in C vs. MDD+/−S (**C**), C vs. MDD−S (**D**), C vs. MDD+S (**E**), and MDD−S vs. MDD+S (**F**) analyses. Each track or ring in the plot represents distinct annotations. The outermost track exhibits hypermethylated sites in red and hypomethylated sites in blue. The second track highlights hypermethylated sites in maroon. The third and fourth tracks display the *p*-value distribution for hypermethylated and hypomethylated sites, respectively.

We determined methylation sites explicitly associated with MDD and suicide among MDD subjects using cross-comparisons of various groups. As shown in Fig. 2A, there were 3693 sites in the control vs. MDD+/−S group, 10,460 sites in the control vs. MDD−S group, 12,295 sites in the control vs. MDD+S, and 33,129 sites in MDD−S vs. MDD+S comparisons. Distinct sets of methylation sites were identified that were specific to MDD and suicide groups. For instance, 30,566 sites were uniquely associated with MDD, while 47,449 sites were specifically associated with MDD individuals who died by suicide, with 2399 sites overlapping between MDD and suicide groups (Fig. 2B). The identification of unique methylation patterns in each group provided valuable insights underlying MDD and suicide pathophysiology.

The Circos plots shown in Fig. 2 offer an overview of the spatial arrangement of significant methylation loci across chromosomes, revealing notable clusters of hypermethylated or hypomethylated sites on specific chromosomes associated with MDD and suicide. In C vs. MDD+/−S analysis, highly significant hypermethylated sites were located on chromosomes 2, 6, 10, 11, and 20 (Fig. 2C). The highly significant hypomethylated sites were mapped on chromosomes 3, 4, 5, 7, 8, 14, 17, and 20. For C vs. MDD−S, significant hypermethylated sites were mapped on chromosomes 1, 3, 4, 15, and 21. Similarly, significant hypomethylated sites were located on chromosomes 1, 2, 3, 4, 8, 10, 12, 16, and 20 (Fig. 2D). Comparison of C vs. MDD+S analysis identified significant hypermethylated sites on chromosomes 1, 2, 3, 4, 6, 7, 9, 11, and 12. Similarly, significant hypomethylated sites were located on 1, 2, 3, 4, 8, 10, 12, 16, and 20 (Fig. 2E). MDD−S vs. MDD+S comparison analysis showed significant hypermethylated sites on chromosomes 3, 5, 6, 10, 11, 17, and 20 and hypomethylated sites on chromosomes 3, 7, 8, 10, and 12 (Fig. 2F). We also mapped the relative position of the top 50 hyper and the top 50 hypomethylated sites across 22 autosomes using PhenoGram with significant differentially methylated (*p* < 0.01) sites on respective chromosomes as determined across the four comparisons (Supplementary Fig. S4).

The Pi diagrams in Supplementary Fig. S5A–H delineated the distribution patterns of methylation sites within genomic regions such as transcription start sites (TSS), exons, CpG islands, and their flanking regions (shores and shelves). Analysis from C vs. MDD+/−S identified a significant percentage of methylation sites located in TSS (31.1%), 5’UTR (20.9%), 3’UTR (14.3%), and exonic (3.7%) regions (Supplementary Fig. S5A). Regarding C vs. MDD−S analysis, the largest number of significant sites were distributed in TSS (28.9%) and 5’UTR (20.1%) regions; the remaining were distributed in exonic (3.5%) and 3’UTR (15.7%) regions (Supplementary Fig. S5B). Interestingly, C vs. MDD+S analysis showed that the greatest number of significant sites were distributed in TSS (29.6%) and 5’UTR (18.5%) regions and the remaining were found in the exonic (2.9%) and 3’UTR (15.5%) regions (Supplementary Fig. S5C) of the genome. MDD+S vs. MDD−S analysis demonstrated the most variability in distribution. The significant sites were distributed in TSS (28.6%) and 5’UTR (19.2%) regions. The remaining sites were found in the exonic (3.2%) and 3’UTR (15.8%) regions (Supplementary Fig. S5D) of the genome.

CpG islands analysis showed the highest CpG density in the island region (48.6%) followed by shore and shelf regions including S_Shore (14.7%), N_Shore (17.15%), S_shelf (5.45%) and N_shelf (6.1%) when C group was compared with MDD+/−S group (Supplementary Fig. S5E). In C vs. MDD−S analysis, only 23.5% CpG islands were in the island region, and others were distributed near S_Shore (8.85%), N_Shore (10.36%), S_shelf (2.97%) and N_shelf (3.14%) (Supplementary Fig. S5F). As with the other two comparisons, CpG island-related distribution of methylation sites from C vs. MDD+S analysis showed that most sites were distributed in the islands region (54.57%), followed by other regions, including S_Shore (14.45%), N_Shore (16.9%), S_shelf (3.89%) and N_shelf (4.16%) (Supplementary Fig. S5G). CpG density analysis from MDD−S vs. MDD+S pair again identified major methylation level changes in the island region (34.9%) followed by S_Shore (9.45%), N_Shore (11.44%), S_shelf (2.7%) and N_shelf (3.04%) regions (Supplementary Fig. S5H). Altogether, the data shows that the highest CpG density occurs in the CpG islands; however, they differ in each analysis, and the most were found in the C vs. MDD+S comparison.

### Gene annotation based on differential methylation sites

**Fig. 3:**
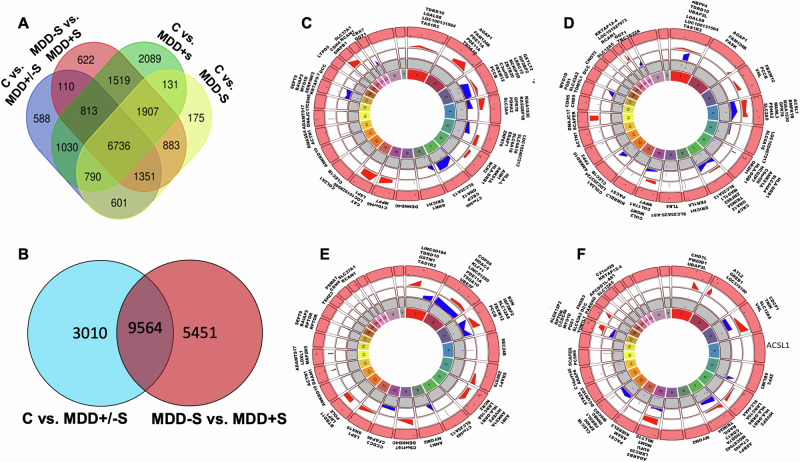
Venn diagrams and circular plots illustrating the distinct and overlapping sets of genes associated with differentially methylated sites (DMs) and their chromosome-wise annotation across the genome in four sets of comparisons. In panel **A**, the focus is on highlighting the unique genes in proximity to the methylation sites identified when comparing DMs individually obtained from analyses contrasting C vs. MDD+/−S, C vs. MDD−S, C vs. MDD+S, and MDD+S vs. MDD−S. Panel **B** depicts a Venn diagram showcasing the unique and shared genes in the vicinity of methylation sites resulting from the differential methylation analysis conducted between C vs. MDD+/−S and MDD+S vs. MDD−S. In panels, **C**–**F**, Circos plots illustrate the chromosome-wise distribution of the most significant differential methylation site-associated genes following a set of comparisons as mentioned in Fig. 2C–F (C vs. MDD+/−S [**C**], C vs. MDD−S [**D**], C vs. MDD+S [**E**], and MDD−S vs. MDD+S [**F**]). Like the previous circular plots, each track represents different annotations. The outermost track showcases the genes associated with hyper- and hypomethylated sites. The next concentric track displays hyper-methylated genes in maroon. The second track highlights the *p*-value distribution (red) for hypermethylated genes. The third track shows the *p*-value distribution (blue) for hypomethylated genes. The fourth track displays hypomethylated genes in a blue color. The innermost circle represents chromosome number.

Gene annotation analyses identified distinct sets of genes exhibiting differential methylation patterns across study groups. A total of 175 genes were implicated in all MDD subjects (MDD+/−S); 588 genes specifically to MDD without suicide (MDD−S); 622 genes to MDD with suicide (MDD+S); and 2089 genes specifically with suicide among MDD individuals (MDD−S vs. MDD+S) (Fig. 3A). To further examine unique and overlapping genes associated with MDD and suicide, we compared C vs. MDD+/−S and MDD+S vs. MDD−S groups. As can be seen in Fig. 3B, 3010 genes were associated with MDD (MDD−S), whereas 5451 genes were associated with suicide among MDD subjects (MDD+S). On the other hand, 9564 genes were common between MDD+S and MDD−S groups.

We also annotated nearby gene loci associated with significant differentially methylated sites and mapped them along with *p*-value distribution on Circos plots. In C vs. MDD+/−S comparison, highly significant hypermethylated genes were found on chromosomes 2, 4, 10, 11, and 20 and highly significant hypomethylated genes were tracked on chromosomes 3, 4, 5, 7, and 17 (Fig. 3C). In the C vs. MDD−S comparison, highly significant hypermethylated genes were mapped on chromosomes 1, 3, 4, 15, and 21, and highly significant hypo-methylated genes were located on chromosomes 1, 2, 3, 4, 8, 10, 12, 16 and 20 (Fig. 3D). For C vs. MDD+S analysis, the hypermethylated genes were mapped on chromosomes 1, 2, 3, 4, 6, 7, 9, 11, and 12, while significantly hypo-methylated genes were located on chromosomes 1, 2, 3, 4, 8, 10, 12, 16, and 20 (Fig. 3E). Lastly, for MDD+S vs. MDD−S analysis, highly significant hypermethylated genes were located on chromosomes 1, 2, 3, 6, 7, 10, and 17, and highly significant hypomethylated genes were located on chromosomes 3, 7, and 12 (Fig. 3F).

### Functional enrichment of genes associated with methylation sites

**Fig. 4:**
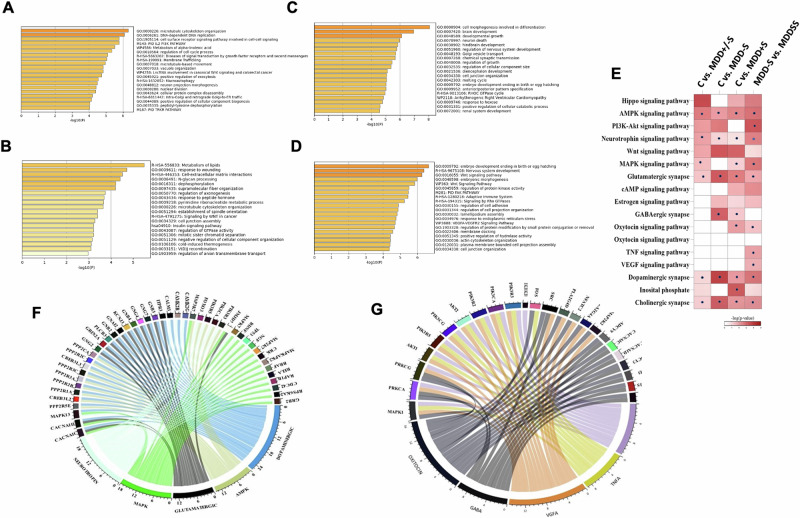
Ontological clustering and pathway mapping of genes associated with differentially methylated sites (DMS). In panel **A**–**D** The top 20 significant gene ontology (GO) terms are visualized in a bar plot, representing four analyses, respectively (C vs. MDD+/−S, C vs. MDD−S, C vs. MDD+S, and MDD−S vs. MDD+S). **E** The dot-heatmap displays statistically significant pathways (*p* < 0.05) identified through the DAVID gene enrichment analysis across four pairwise comparisons. Each row corresponds to a pathway, with blue dots indicating pathways where the same genes were found in more than one analysis, thereby highlighting recurrent pathways. The color intensity of each dot corresponds to the -log *p*-value, with darker shades indicating higher statistical significance. Pathways that consistently appear across multiple analyses are highlighted, with these recurring patterns reinforcing our confidence in their potential biological importance. In panels, **F**, **G**, the functional clustering of methylated genes and their relationships are illustrated using a chord diagram. This diagram depicts pairwise comparisons between C vs. MDD+/−S and MDD−S vs. MDD+S, respectively.

To identify the functional correlation of significant methylation sites and nearby genes across the genome, we first determined the proximal genes of the top 50 hyper and 50 hypomethylated sites across various comparisons. The list of identified genes from each pair of analyses is provided in Supplementary Table S11. With the help of gene function enrichment analysis tools, gene ontological clusters were determined, and the top 20 significant GO terms are presented in bar plots from four analyses (Fig. 4A–D). For C vs. MDD+/−S comparison, neuron projection, morphogenesis, and regulation of the cell cycle process (Fig. 4A), whereas for C vs. MDD−S, metabolism of lipids and cell-extracellular matrix were the key pathways (Fig. 4B). C vs. MDD+S comparison gave gene function enrichment for brain development and neuron death (Fig. 4C). On the other hand, MDD+S vs. MDD−S analysis highlighted nervous system development, Wnt signaling pathway, and cellular process (Fig. 4D). In Fig. 4E, the key pathways from all four analyses were compared to highlight those that repeatedly appeared across methods. Pathways marked with a blue dot have genes identified in more than one analysis, indicating their relevance and participation in critical biological processes. The color intensity, indicating -log *p*-value, shows the statistical significance-darker shades denote higher confidence in that pathway’s relevance. Together, blue dots and darker colors highlight pathways that stand out both in recurrence and statistical strength. Next, we functionally clustered differentially methylated genes to determine associated key pathways. The key pathways in four different comparisons are listed in Supplementary Table S12. From C vs. MDD+/−S analysis, neurotrophin, MAP kinase, glutamatergic, AMPK, and dopaminergic pathways were predominant (Fig. 4F). On the other hand, MDD−S vs. MDD+S comparison yielded pathways such as Oxytocin, GABA, VGFA, TNFA, and MTOR (Fig. 4G). In addition to the GO bar plot analysis, we generated gene-gene networks from all four analyses, which represent the overrepresented gene lists. For instance, in the analysis involving individuals with MDD+S, there was a notable enrichment of genes related to Wnt and VEGFA signaling (Supplementary Fig. S6A). Meanwhile, neuro-projection morphogenesis emerged as a distinctive feature when comparing C with MDD+/−S groups (Supplementary Fig. S6B). Conversely, the MDD−S group demonstrates enrichment of genes associated with GTPase signaling pathways (Supplementary Fig. S6C). The comparison between C and MDD+S revealed functional pathways related to neuronal death and synaptic transmission (Supplementary Fig. S6D), uniquely enriched, which was not observed in other analyses.

**Fig. 5:**
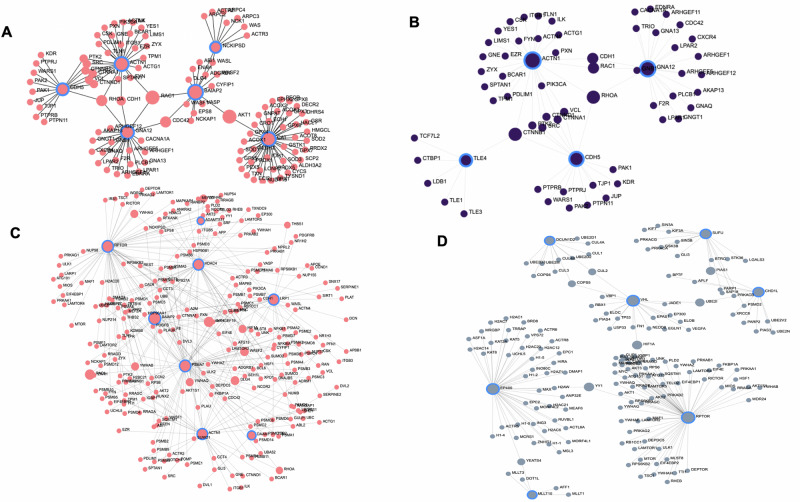
The Protein-Protein Interaction (PPi) network illustrating the comprehensive interconnections among genes closely linked to the top 50 significantly hypermethylated and 50 hypomethylated sites across the genome in the contexts of depression and suicide history. The hub genes connecting each small subnetwork under a central network have been shown with a larger circle for each individual analysis. **A** The PPi network is constructed based on genes identified in the comparison between C vs. MDD+/−S. **B** PPi network generated using genes derived from the comparison between C vs. MDD−S. **C** PPi network created using genes identified in the comparison between C vs. MDD+S. **D** The PPi network was constructed based on genes derived from the MDD−S vs. MDD+S comparison.

Next, using the protein-protein interaction (PPI) network (genes associated with the top 50 hyper and the top 50 hypomethylation sites), we identified hub genes enriched in brain functions across study groups (Fig. 5A–D), offering a network-based perspective on molecular interactions associated with differential methylation patterns in MDD and suicide. In the C vs. MDD+/−S analysis, we found six hub genes in the network and three sprouting nodes in the subnetworks (Fig. 5A). The four prominent hub genes with the maximum number of branches were CDH5, ACTN1, GNA12, and CAT. The other two hub genes were BAIAP2 and NCKIPSD. In the C vs. MDD−S comparison, we found a relatively smaller PPi network with two major hub genes (ACTN1 and GNA12) and two other hub genes (CDH5 and TLE4) with relatively smaller branching of edges (Fig. 5B). ACTN1 and GNA12 as hub genes were also identified as central hubs in the C vs. MDD+/−S comparison. In the C vs. MDD+S analysis, the largest PPi network was identified with seven hub genes (Fig. 5C). They were: RAPTOR, HDAC4, ADAMTS17, HSP90AA1, PSMA7, DAAM, LRP1, and ACTN1. All these hub genes had the maximum number of edges among all four comparisons. In MDD−S vs. MDD+S, a moderate-size PPi network was mapped with two central hub genes, EP400 and RAPTOR (Fig. 5D). Interestingly, EP400 and RAPTOR formed a quasi-network, loosely connected with two other subordinate networks central to the hub genes, i.e., VHL, DCUN1D2, SUFU, and CHD1L. All hub genes from the four comparisons are summarized in Supplementary Table S13.

### Annotation of differentially methylated genes (DMG) for tissue specificity and regulatory elements

Tissue-specific expression analysis using the GTEx database confirmed brain-specific enrichment of key genes involved in differential methylation, highlighting their potential roles in regulating brain function and behavior. The analysis demonstrated that identified genes exhibited preferential expression in brain tissues, emphasizing their relevance in the context of MDD and suicidal behavior. All key genes from GO-based functional enrichment analysis (VEGFA, PTK2, ULK3, PRKCG, ERBB2, GABBR1, ATP6V0B, and BRAF) had tissue specificity related to different brain regions (Supplementary Fig. S7).

### Differential expression analysis of functionally enriched genes in MDD subjects with and without suicide

qPCR-based expression data showed significant decreases for VEGFA and BRAF across all three groups (MDD+S, MDD−S, and total MDD) compared to the control group. On the other hand, PTK2, ULK3, PRKCG, GABBR1, ERBB, and ATP6V0B showed a significant trend in expression upregulation across all three groups compared to the control group. Interestingly, the highest expression increase (*p* < 0.05) was noticed for the ERBB2 gene in the MDD−S group (~7-fold), followed by upregulation in MDD+S and total MDD groups. The expression changes are shown in Supplementary Fig. S8.

### Effects of confounding variables on specific methylation sites

As shown in Supplementary Table S14, age, PMI, RIN, and brain pH were not significantly correlated with the top 25 significantly methylated sites in the MDD group. Similarly, antidepressant toxicology, race, and sex had no significant effects on these significantly altered methylated sites except for site 18, where Black MDD subjects showed significantly higher methylation than White MDD subjects (Supplementary Figs. S9–11).

## Discussion

Both Major Depressive Disorder (MDD) and suicide are polygenic and involve complex gene-environment interactions [32–34], requiring a system-wide approach to understand their molecular pathologies [35]. So far, research on DNA methylation has been focused on depressed suicide subjects [20, 36, 37], not providing suicide-specific epigenetic marks in MDD subjects. This study aimed to distinguish the epigenetic landscape between MDD and suicide brains and determine if specific DNA methylation changes increase suicide risk among MDD patients. Our findings show significant differences in DNA methylation at numerous CpG sites in the dlPFC of MDD subjects who died by suicide compared to those who died from other causes; however, a significant proportion of methylated CpGs were also common between the two groups. Our initial PCA analysis did not find any specific effects of age, race, or sex, as no specific DNA methylation clusters were found among control and MDD subjects. Next, in the analysis, we combined differentially methylated regions with functional genes in the vicinity. Bioinformatic analysis of differentially methylated regions, including specific CpGs sites, identified genes connected through various functional networks. The data suggested the relatedness of some of the key genes as hubs to regulate the functions of other genes that are topographically positioned in the periphery of the network [38, 39]. The functional relatedness of these methylation-associated genes further helped us to translate their ontological roles in neurobiological pathways such as stress adaptation, inflammatory cascade, metabolic processes, transcription factors, and neurotransmission [36, 38, 40].

Genome-wide DNA methylation changes are increasingly recognized as playing a role in the clinical manifestation of MDD [41]. Studies have identified altered methylation patterns in genes involved in key pathways such as stress response, neuroplasticity, and immune function, which are relevant to MDD [42, 43]. For instance, hypermethylation of the SLC6A4 gene promoter has been associated with reduced serotonin availability [44, 45]. The SLC6A4 gene is also responsible for serotonin transport, and its reduced availability in the brain is a classical hallmark of MDD [46]. Additionally, changes in the methylation of the BDNF gene have been implicated in MDD and suicide [47–49]. Lower expression of BDNF is linked to impaired synaptic functions in MDD [50]. Environmental factors like early-life stress have also been shown to induce long-lasting DNA methylation changes, potentially increasing susceptibility to depression [51, 52]. Collectively, these findings suggest that DNA methylation marks on specific genomic loci could serve as potential biomarkers for MDD [53–55]. At the same time, they may offer targets for novel therapeutic approaches [56]. Additionally, their screening in peripheral circulation as cell-free DNA with intact methylation marks could be of interest in MDD-based biomarker development [57]. Our study observed similar hypermethylation patterns in other genomic loci, which could further support their role in MDD pathophysiology. These results reinforce the link between epigenetic modifications and the molecular mechanisms underlying MDD. Interestingly, we observed dysregulation and impairment of several cellular pathways related to key changes in synaptic and neurotrophic functions. Moreover, our research uncovered novel methylation changes in additional genomic regions, including the genes from oxytocin, GABA, VGFA, TNFα, and mTOR signaling pathways [35]. The impact of methylation of these genes needs to be studied further in the context of MDD. Nevertheless, these findings expand the current understanding of the epigenetic landscape of depression, suggesting that other biological pathways may be involved in the disorder’s manifestation. We observed that adjacent methylation changes near the sites of Wnt signaling pathway genes might have a significant role in associating the methylation marks with an increased risk of suicide [58, 59]. By identifying well-established and novel methylation markers, our study contributes to the growing evidence of DNA methylation’s role in MDD pathogenesis. These findings offer new opportunities for exploring DNA methylation as a potential biomarker for diagnosis and as a target for developing more personalized therapeutic strategies for depression.

We examined site-specific genome-wide DNA methylation patterns in various combinations. Initially, all MDD subjects were combined (with and without suicide; MDD+/−S) and compared with controls. Subsequently, MDD without suicide (MDD−S) and MDD with suicide (MDD+S) groups were compared with the control group independently. Additionally, the MDD+S group was compared with the MDD−S group. In the MDD+S vs. MDD−S comparison, we mapped the highest number of differential methylation sites (49848) spanning the maximum number of genes (15015) across the genome. On the contrary, the lowest methylation density was noticed in the control vs. MDD−S analysis, where 30852 methylation sites were mapped across 12019 genes. In the MDD−S vs. MDD+S comparison, we identified three hypermethylated positions (cg07167933, cg04842790, and cg26106837), which reached the experiment-wide significance (*p* < 0.05). These hypermethylated probes were located in close proximity to genes that were found to be part of oxytocin, GABA, VGFA, TNFα, and mTOR signaling pathways. All these signaling pathways have been reported not only to be critically involved in various brain functions but also in MDD and, to a certain extent, suicidal behavior [60–65]. We also determined the differential methylation profile in MDD subjects compared to controls and were able to map three probes (cg18037388, cg00892228, cg11866463) that showed the highest significance level in the MDD group. Functional clustering of the genes nearby to these hypermethylated probes determined pathways, including neurotrophin, MAP kinase, glutamatergic, AMP kinase, and dopaminergic systems. All these pathways are known for their critical role in neurogenesis and plasticity [35, 66]. The protein-protein interaction network analyses based on the genes that were in close proximity with the top 100 significantly hyper and hypomethylated sites provided insights into their roles in the molecular pathogenesis of MDD and suicide. For instance, three hub genes, CDH5, ACTN1, and GNA12, were common in MDD suicide and MDD non-suicide subjects, which could activate or repress an array of gene sets within the network (CAT, BAIAP2, NCKIPSD, RAPTOR, HDAC4, ADAMTS17, HSP90AA1, PSMA7, DAAM, LRP1) with specific roles to develop endophenotypes associated with depression and suicide [62, 67]. Further examination showed regulatory functions associated with these genes. For example, CAT is involved in innate immunity, BAIAP2 in neurite growth and maintenance of dendritic spines, NCKIPSD in modulation of synaptic activity in neurons, RAPTOR in excitatory neuronal activity, ADAMTS in neuronal plasticity and cognitive impairment, PSMA7 in inflammatory immune response, DAAM in axonal growth cone formation and regeneration, HDAC4 in activity-dependent synaptic transmission, and HSP90AA1 in HPA axis-related neuroendocrine abnormalities [68–72]. We found the role of RAPTOR and ADAMTS quite interesting, given their regulatory roles in mTOR-mediated synaptic reorganization and extracellular matrix pathways [73, 74]. Recent studies have suggested the role of the mTOR pathway in repairing cytoskeletal organization and inducing AMPA receptor functions in frontocortical regions [75, 76]. Thus, epigenetic silencing of genes like ADAMTS17 or RAPTOR may compromise mTOR and AMPA receptor-mediated synaptic plasticity and can increase suicide vulnerability among MDD patients [65, 77, 78].

Chromosomal localization of the methylation sites across group-wise comparisons showed a high prevalence on chromosomes 2, 3, and 4. All three chromosomes were mapped for a maximum number of genes with differential promoter methylation states. The key distribution of hyper- and hypo-methylated genes on these three chromosomes may have a role in the increased risk of MDD and suicide [79, 80]. Further examination showed that hypermethylated genes were preferably mapped on chromosome 2, whereas chromosome 3 was largely found with hypomethylated genes. We anticipate that a further fine-scale genetic association map could help us provide a genetic framework of the methylation-sensitive genes on these three chromosomes.

Our gene enrichment analysis provided the potential roles of hypermethylated sites in regulating the active and repressive elements in relation to MDD and suicide pathophysiology. A comparison of the DNA methylation differences with genomic annotations showed that these differentially methylated regions were enriched at TSS, 5’UTR, 3’UTR, and exon regions. In the control vs. MDD+/−S analysis, a significant percentage of methylation sites were located close to TSS (31.1%), which was by far the largest proportion of TSS-associated methylation sites among various comparison groups. The second largest proportion (29.64%) was detected in the control vs. MDD+S analysis. This suggests that higher promoter occupancy by CpG methylation in MDD subjects may be associated with genes with an immediate effect on their expression downregulation [81, 82]. This could be part of maladaptive changes in the MDD brain, making the underlying molecular circuitry vulnerable to an increased risk of suicide. This can be clearly noted in the control vs MDD−S analysis, where the CpG island distribution in the TSS area was reduced by 28%. Less variability was noted in the exonic and 3’UTRs across all comparisons [83]. This follows the principle of DNA methylation changes at the promoter area with a repressive regulatory role in gene expression [84]. It has previously been shown that cytosine-based DNA methylation changes in gene bodies are mostly associated with expression upregulation [85]. In addition, our CpG site distribution analysis showed the highest differentially methylated sites in the island region in control vs. MDD+S analysis. This indicates that methylation levels of CpG islands were higher than CpG island shores in the promoter, exon, or intron regions in MDD suicide subjects. These results demonstrate that CpG islands with different genetic features display differential effects on the methylation patterns of the associated genes and are transcriptionally repressive [86, 87]. It is interesting to note that the shores and shelves of the islands were found to be modestly methylated across all comparisons in this study. Moreover, our gene function enrichment analysis, supported by tissue-specific database information, highlighted some of the genes enriched in brain functions. We further selected eight key genes for expression analysis using the same brain samples we used in global methylation analysis. The eight genes used in this expression profiling are based on our prediction-based GO enrichment analysis and might hold potential clues to their roles in MDD and suicidal pathogenesis. As previously shown, PTK2, ULK3, PRKCG, GABBR1, and ATP6V0B play significant roles in the neurobiological mechanisms of neuroplasticity, autophagy, and neurotransmission [88–91]. In particular, VEGFA, ERBB2, and BRAF play significant roles due to their involvement in inflammation, and cellular signaling [92, 93]. For example, VEGFA is known for its role in angiogenesis, and any disruption in gene function can cause maladaptivity in neurogenesis and synaptic plasticity [94]. Studies have found decreased VEGFA levels in the MDD brain [95]. The depletion in VEGF level has also been associated with disruptions in VEGFA signaling, causing neurovascular damage to the MDD brain and conferring an increased risk of suicidality [96]. Our previous study also identified VEGFA as a target of miRNA in the MDD brain, revealing how miRNA expression changes in depression and suicide may influence VEGFA and related neuro-epigenetic pathways [97]. Additionally, the VEGFA is responsive to antidepressant treatment. For example, stress-induced inhibition of VEGFA expression in the brain is blocked by antidepressant treatment. The action of antidepressants in removing the blockage on adult neurogenesis has been shown to be mediated through the CREB response pathway, which acts on VEGFA signaling [98]. Another gene, ERBB2, is crucial for neurodevelopment and neuronal survival via the Neuregulin, PI3K/AKT and MAPK pathways [99]. Evidence suggested that disruptions in ERBB2 signaling may lead to neuroinflammatory responses and stress-induced neurotoxicity [100]. Interestingly, both these changes are related to MDD and suicide risk [101]. BRAF, a part of MAPK/ERK pathway, supports cell survival, differentiation, and synaptic plasticity [102]. Interestingly, impaired ERK signaling has been found to be associated with reduced catalytic activity and protein expression of BRAF in the suicide brain [103]. Moreover, reduced levels of BRAF have been found in bipolar disorder brains [93]. These findings collectively indicate that BARF may exacerbate stress response, influencing mood and suicidal behavior [93]. Altogether, these genes underscore neurogenetic, inflammatory, and signaling pathways implicated in MDD and suicide, highlighting potential therapeutic targets.

Lastly, we determined similar and unique sites across four pairs of analyses and identified 30560 unique methylation sites from C vs. MDD+/−S and 47449 from MDD+S vs. MDD−S analyses. Along with methylation sites, we also identified unique 3010 genes from C vs. MDD+/−S and 5451 from MDD+S vs MDD−S analyses. Functional clustering of the genes determined key biological processes from C vs. MDD+/−S analysis yielded neuron projection, morphogenesis, and regulation of cell cycle process, and MDD+S vs. MDD−S analysis highlighted nervous system development, Wnt signal pathway, and cellular process. On the other hand, in control vs. MDD+S analysis, we noted gene function enrichment for brain development and neuronal death. Previous reports have suggested a significant role of β-catenin, a component of Wnt signaling, in MDD [104]. β-catenin is regulated by neurotransmitters such as serotonin and dopamine, and any fluctuations in β-catenin under stressful conditions can lead to depression-like behavior [104, 105]. On the other hand, increased expression of Wnt2 ligand can have antidepressant effects [106]. Abnormality in Wnt signaling has also been noted in the brains of suicide subjects [107].

Altogether, our genome-wide epigenetic study in the dlPFC found differentially methylated regions in MDD subjects, which may influence genes involved in synaptic reorganization, immune response, and neurogenesis [35, 108, 109]. Also, our data indicates significant methylation differences in MDD individuals who died by suicide or causes other than suicide. Additionally, we found suicide-specific methylation changes when the MDD−Suicide group was compared with the MDD-non-suicide group, suggesting that specific methylation changes may be associated with the risk of suicide among MDD individuals. These methylation changes could potentially be used to diagnose MDD individuals who are at risk of suicide.

While our study delivers a clear understanding of genome-wide methylation changes in MDD and how it could be associated with an increased risk of suicide, there are a few limitations related to this study. For example, although our findings are quite robust, even with a limited number of subjects, it underscores the need for future studies with a larger sample size. Additionally, probe-based DNA methylation arrays have restricted coverage because they are designed based on pre-selected CpG sites and are often limited to CpG islands near promoter elements. Due to this site selection, some methylation changes in non-genic regions like introns may remain undetected.

## Supplementary information

Suypplementary section

Supplementary Table 3

Supplementary Table 4

Supplementary Table 5

Supplementary Table 6

Supplementary Table 7

Supplementary Table 8

Supplementary Table 9

Supplementary Table 10

Supplementary Table 11

Supplementary Table 12

Supplementary Table 13

Supplementary Table 14

## Data Availability

All data generated or analyzed during this study are included in this published article [and its supplementary information files].
