# Supplementary material for: Genome-wide methylome-based molecular pathologies associated with depression and suicide
Source: Neuropsychopharmacology. 2024 Dec 7;50(4):705–16. doi: 10.1038/s41386-024-02040-9 (PMC11845511; doi:10.1038/s41386-024-02040-9)
Supplement: Supplementary file 1 — Suypplementary section [file 41386_2024_2040_MOESM1_ESM.pdf]

SUPPLEMENTAL SECTION

Genome-wide methylome-based molecular pathologies associated with depression and suicide

Yogesh Dwivedi, Ph.D.[1,*] Bhaskar Roy, Ph.D.[1], Praveen Kumar Korla, Ph.D.[1]

Department of Psychiatry and Behavioral Neurobiology, University of Alabama at Birmingham, Birmingham, Alabama, 35294, USA

[*]Corresponding author:

Yogesh Dwivedi, Ph.D.
Distinguished Professor of Psychiatry
Elesabeth Ridgely Shook Endowed Chair for Psychiatric Research
Director of Translational Research, UAB Mood Disorder Program
Co-Director, UAB Depression and Suicide Center
Department of Psychiatry and Behavioral Neurobiology
University of Alabama at Birmingham
SC711 Sparks Center
1720 7th Avenue South
Birmingham, Alabama, USA
Phone: 1-205-975-8459
Email: yogeshdwivedi@uabmc.edu

SUPPLEMENTAL METHODS

Human Postmortem Brain samples

The study was approved by the Institutional Review Board of the University of Alabama at Birmingham. Samples from dlPFC were obtained from the Maryland Brain Collection at the Maryland Psychiatric Research Center, Baltimore, MD. The cohort comprised 48 brain samples, among which 15 were MDD subjects who died by suicide (MDD+S), 17 were MDD subjects who died by causes other than suicide (MDD-S), and 16 were control subjects without psychiatric disorders (referred to as controls [C]). For analysis purposes, we created an additional group of subjects combining all MDD subjects with and without suicide (MDD+/-S). Tissues were collected only after a family member gave informed consent. All tissues from controls and MDD subjects were screened for evidence of neuropathology and were excluded if they exhibited features of Alzheimer's disease, infarctions, demyelinating diseases, or atrophy (or clinical history of these disorders). Toxicology and the presence of antidepressants were examined by analysis of urine and blood samples from these subjects. Brain pH was measured as described previously. Detailed demographic and clinical characteristics of subjects are shown in **Table S1**. The psychiatric diagnosis was determined by psychological autopsy as described earlier (27) using Diagnostic Evaluation After Death (DEAD) (28) and the Structured Clinical Interview for the DSM-V (SCID) (29). The interviews were done by a trained psychiatric social worker. Two psychiatrists independently reviewed the write-up from this interview, as well as the SCID that was completed from it, as part of their diagnostic assessment of the case. Diagnoses were made from the data obtained in this interview, medical records from the case, and records from the Medical Examiner's office. The two diagnoses were compared and discrepancies were resolved by means of a consensus conference. Control subjects were verified as free from mental illnesses using these consensus diagnostic procedures.

After removal from the cranium, the brains were cut into six major pieces (four cerebral cortical lobes, basal ganglia-diencephalon, and lower brain stem-cerebellum), rapidly frozen on dry ice, and stored at −70°C until dissection. During dissection, the frontal lobes were sliced into 1-mm to 1.5-mm thick coronal sections at a temperature between 0°C and 10°C. To keep the samples frozen, the dissections were performed on a metal plate over a container filled with dry ice. The prefrontal cortical samples were cut out of the coronal sections by a fine microdissecting (Graefe) knife under a stereomicroscope with low magnification. The dorsomedial prefrontal cortex (Brodmann's area 9) was taken just dorsal to the frontopolar area, including the most polar portion of the superior and partly the middle frontal gyrus between the superior and intermediate frontal sulci. In the sections of the dissected cortical area, the gray and white matter were separated. The tissues were chopped into smaller pieces and stored at −80°C until use.

All tissues from control subjects and suicide victims were screened for evidence of neuropathology by experienced neuropathologists at each brain collection program. The tissues were examined histologically. Fixed sections of PFC were screened with hematoxylin and eosin (H&E) staining and an antibody to glial fibrillary acid protein. Alzheimer’s disease, infarcts, demyelinating diseases, or atrophy disqualified subjects from the study. In addition, in each case, screening for the presence of human immunodeficiency virus (HIV) was done in blood samples, and all HIV-positive cases were excluded. Toxicology data were obtained by the analysis of urine and blood samples.

DNA methylome assays with Illumina 850K Infinium methylation EPIC array

Genomic DNA was extracted from dlPFC using a QIAamp ® genomic DNA extraction kit (Qiagen, Germany). The concentration of extracted genomic DNA was measured with Nanodrop (Thermo Scientific, USA), and the OD260/OD280 ratio was used for detecting any residual

contaminants in DNA preparation. Isolated DNA samples were treated with bisulfite reagents following the manufacturer's protocol using a DNA methylation kit (Zymo Research, USA). Treated DNA samples after purification were then hybridized with the 850K Infinium Methylation EPIC BeadChip (Illumina Inc., USA), which covered over 850 000 methylation sites located in CpG islands, genes, transcription binding sites, open chromatin regions, and enhancers at single-nucleotide resolution. Both unmethylated and methylated CpGenome controls were included, and duplicates of a pooled DNA sample were incorporated in each array to assess inter-array consistency. The Illumina iScan was employed to scan the arrays, capturing high-resolution images of the light emitted by the excited fluorophores at each CpG site. The raw intensity data from these images were stored as .idat files for subsequent analysis. For processing these .idat files, the Illumina Genome-studio 2.0 software methylation module was utilized. Each cohort was independently analyzed, applying the same quality control (QC) and pre-processing pipelines. We then performed functional normalization, a critical process that standardized the signal intensities and, at the same time, accounted for technical differences between arrays. These are essential steps to reduce unwanted variability and ensure the robustness and consistency of our methodology across samples. Separate background adjustments of methylated and unmethylated intensities of type I and II probes were also conducted at the same time. The quantile normalization method was employed to equalize the total signal intensities and distributions of probe signal strengths among arrays or among color channels on an array. It sorts all probes by signal strength and then matches probes at each rank position among arrays and forces the values at each rank position to be equal. An identical distribution of probe signal strengths among the arrays or color channels was obtained. Next, all data files were imported into R and analyzed using the ChAMP package (https://bioconductor.org/packages/release/bioc/html/ChAMP.html). Champ is a comprehensive

R/Bioconductor pipeline designed to read raw data from Illumina IDAT files, perform filtering and quality control, and carry out all subsequent analyses. Following the processing steps, probes were excluded if they had a detection p-values were greater than 0.05. Additionally, all probes on the X and Y chromosomes were filtered out. The normalized intensity signals were converted into DNA methylation levels and presented as beta(β)-values. The champ.DMP function was employed to identify the differentially methylated probes (DMP) and champ.DMR with the Bumphunter method was used to identify differentially methylated regions (DMR) across the study groups, including control, MDD-S, and MDD+S. Collectively, these steps allowed us to determine differential methylation changes and helped us reach meaningful conclusions regarding the epigenetic differences between the three groups.

For the statistical analysis of differentially methylated sites, we followed an unpaired t-test for normally distributed data, comparing the two groups. To account for multiple tests, we applied the False Discovery Rate (FDR) correction using the Benjamini-Hochberg procedure, with an FDR threshold of 0.05. Additionally, we considered an absolute difference in beta values greater than 0.2 as biologically significant.

Methylation data pre-processing

Probes with a detection level of $p > 0.05$, non-specific probes, potentially cross-reactive probes, or probes near SNPs 35 and 36 were removed across all samples. For the annotation of probes, the human (GRCh37/hg19) assembly was used (30). To further ensure consistency at the data extraction level, raw DNA methylation data (i.e., idat files) were recovered, and each cohort was independently analyzed, applying the same quality control (QC) and pre-processing pipelines. Briefly, QC checks, quantile normalization, and separate background adjustment of methylated and unmethylated intensities of type I and II probes were employed using the Genome-studio 2.0

software (https://www.illumina.com/techniques/ microarrays/ array-data-analysis-experimental-design/genomestudio.html).

The final analyses included 813,382 autosomal probes. All samples passed QC procedures. Signal intensities and raw methylation β values were extracted from Illumina's Genome-Studio software R package ChAMP (https://bioconductor.org/packages/ release/bioc/html/ChAMP.html). The methylation β values were generated based on normalized signal intensities and after background subtraction using negative control probes. Methylation β values were derived as the ratio of methylation probe intensity to overall intensity.

Differential methylation data analysis and visualization

In this study, we report four independent analyses to identify differential methylation sites across different groups, as mentioned in the previous section. The complete outline of the methylation analysis following the genomic feature extraction steps is presented with a schematic diagram in **Figure S1**. Each analysis was done to calculate β value differences between the groups based on a threshold level of 0.02 to either determine hyper- (>0.02) or hypomethylated (>-0.02) sites following statistical significance ($p<0.05$). Quantile-Quantile (Q-Q) plots were employed to validate the distributional assumptions of methylation data across comparisons. In the Q-Q plots, the expected values of each methylation site were plotted in red, and the observed values of each methylation site were plotted in black. We also applied feature extraction tools to annotate the significant methylation sites for relative chromosomal localization of methylation site, associated gene name, CpG islands information, and prediction-based functional clustering. Volcano plots were generated with differential methylation (DM) data values. All DM sites in Volcano plots showed a p-value <0.05. The significantly differential methylation sites ($Log_{10}P$ value >3) are labeled with red (hypermethylated) or blue (hypomethylated) color. The hyper- and

hypomethylated sites were also mapped across 22 autosomes using PhenoGram (http://visualization.ritchielab.org/ phenograms/ plot). With this, we visualized the top fifty hypermethylated and fifty hypomethylated sites with blue and green color codes, respectively. To add clarity to the relative methylation sites, we also plotted the gene names that are in the vicinity of the respective methylation sites on individual chromosomes.

Additionally, we used Manhattan (https://jee-hyoung-kim-9.shinyapps.io/ Manhattan_Plot/) to visualize the chromosome-wide distribution of DM sites and represented observed vs expected beta values for the same. In the plot, we included DM sites with log10 p-value >5 and termed them as the most significant DM sites.

Gene ontology term enrichment analysis

The differentially methylated gene lists (p <0.01) extracted from the four group analyses were used in the David functional enrichment analysis tool (https://david.ncifcrf.gov/) to determine the functional enrichment of genes for ontological clustering and pathways. Briefly, pathways were downloaded from the Gene Ontology (GO) website (http://geneontology.org/), and all genes annotated to parent terms were included. Genes containing at least one Illumina probe and annotated to at least one GO pathway were considered. The GO prediction analysis was done following an FDR-corrected p-value cutoff of 0.05 to determine the gene-set enrichment in the biological process (BP) and cellular component (CC) categories separately. In BP, the 30 most significant terms were used to plot the network with an edge cutoff of 0.05, whereas, in the CC, the connected nodes are presented with the 40 most significant terms with an edge cutoff of 0.03. Gene Set Enrichment Analysis results with statistical significance (p<0.05) were applied to determine the key pathways. Metascape software was used to generate GO bar plots, functional gene networks, and protein-protein interaction modules for all four group analyses.

Next, we examined brain-specific expression enrichment of key methylated genes in the context of other tissues. We used the Genotype-Tissue Expression (GTEx) database to study the tissue-specific gene expression. GTEx includes data from 54 non-diseased tissue sites in >1000 individuals. This portal provides not only gene expression data but also QTLs and histology images. Key genes were annotated with GTEx resource data to represent brain tissue specificity.

cDNA synthesis and qPCR

qPCR-based expression profiling of eight genes (VEGFA, PTK2, ULK3, PRKCG, ERBB2, GABBR1, ATP6V0B, and BRAF) were performed in dlPFC and the expression data were compared to determine differential changes in MDD+S (16), MDD-S (n=15) and total MDD (n=31) groups while comparing them with control subjects (n=16). We used 500 ng RNA to synthesize cDNA using M-MLV Reverse Transcriptase (Invitrogen, Grand Island, NY, USA) and oligo (dT) primer as previously described (REF). The relative value of transcripts was measured by a quantitative real-time PCR machine (Stratagen MxPro3005, La Jolla, CA, USA) with 1X Syber Green qPCR mastermix (New England Biolab, MA, USA), 0.8 μM each of gene-specific forward and reverse primers (**Table S2**). Twenty-fold diluted cDNA was used as a template to conduct qPCR. To exclude the possibility of primer dimer formation and secondary product amplification, we conduct further steps of the Syber Green specific dissociation curve analysis program with an initial denaturation at 72°C for 1 min, annealing at 55°C for 30 sec, and repeat denaturation step at 95°C for 30 sec. We selected GAPDH among housekeeping genes as an internal standard. We calculated fold change following Livak's ΔΔCt method. We excluded some values from qPCR results because those values were significant outliers according to the outlier calculator (https://www.miniwebtool.com/outlier-calculator/). For VEGFA and BRAF gene

expression study, we used TaqMan probe chemistry separately purchased from Invitrogen (Invitrogen, USA).

**Table S1: Demographic and clinical characteristics of subjects**				
	**Non-psychiatric Controls (C)**	**Total MDD (MDD+/-S)**	**MDD+S**	**MDD-S**
Number of subjects	16	32	15	17
Age (Year)	36.68±11.02	45.25±16.45 (F=2.51, p=0.12, t=-1.87, df=46)	50.66±15.55 (F=1.06, p=0.31, t=-2.90, df=29)	40.47±16.15 (F=1.3, p=0.26, t=-0.78, df=31)
PMI (Hours)	17.37±5.66	18.75±6.89 (F=,0.49 p=0.48, t=-0.69, df=46)	17.93±8.73 (F=3.71, p=0.06, t=-0.21, df=29)	19.47±4.90 (F=0.70, p=0.41, t=-1.13, df=31)
RIN	7.25±0.32	7.48±0.29 (F=1.21, p=0.28, t=0.68, df=46)	7.45±0.25 (F=2.29, p=0.14, t=0.87, df=29)	7.5±0.33 (F=0.40, p=0.53, t=0.34, df=31)
Brain pH	7.25+0.20	7.27±0.23 (F=0.12, p=0.73, t=-0.28, df=46)	7.32±0.26 (F=0.003, p=0.95, t=-1.00, df=29)	7.21±0.22 (F=0.44, p=0.51, t=0.44, df=31)
Gender				
Males	10	20	10	10
Females	6	12	5	7
Cause of Death	Pneumonia, ACI, ASCVD, CA, MVA, hemopericardium, embolism		GSW, jumped from height, hanging, CO intoxication, drug overdose, stab wound	ASCVD, MVA, ketoacidocis, cardiomegaly, seizure, fatty liver
Race	1 Black/15 White	4 Black/1 Asian/27 White	1 Black/1 Asian/13 White	3 Black/14 White
Neurological/ Neuropathological disorders	None	None	None	None
Number of subjects showing positive antidepressant toxicology	None	10	3	7

Number of subjects showing substance use	None	2	None	2
ACF, Acute cardiac failure; ACI=accidental chest injury; AMI, acute myocardial infarction; ASCVD, atherosclerotic cardiovascular disease; CA, cardiac arrhythmia; MVA, multiple vehicle accident; Data are the mean ± standard deviation; The data were analyzed using Independent Sample Test. Data were compared with control group.				

**Table S2: Gene expression primer sequences**	
Oligo Name	Sequence (5'-3')
hsa_VEGF RT F	AAGGAGGAGGGCAGAATCAT
hsa_VEGF RT R	TGGCCTTGGTGAGGTTTGAT
hsa_PTK2 RT F	AGGGAGCAGGAACATCAGTG
hsa_PTK2 RT R	CTGATCCCTGTTGTTGCTGC
hsa_ULK3 RT F	TGTCCTTGCTCCTGCTATCC
hsa_ULK3 RT R	CAGGCTCTGATCTTCAGCCA
hsa_PRKCG RT F	TGAGGAGGGCAAGATCTGAC
hsa_PRKCG RT R	TTGGGAGGGTCAGAGTGTTT
hsa_ERBB2 RT F	CGGCTCTATGGAAGAGTGCT
hsa_ERBB2 RT R	CAGCTTCTGCTTCTCCACCA
hsa_GABBR1 RT F	CTGGAAGTGGGAGGCTTACA
hsa_GABBR1 RT R	GACCTTCCAGAGCTCTCAGC
hsa_ATP60B RT F	TGGCGGATTTCGTATGTGAA
hsa_ATP60B RT R	CAGGAAGGAGGGAGGATGAT
hsa_BRAF RT F	AGGAACCTGTTTTCCTGGGC
hsa_BRAF RT R	CTGTTGAGCAGCATCTCTC

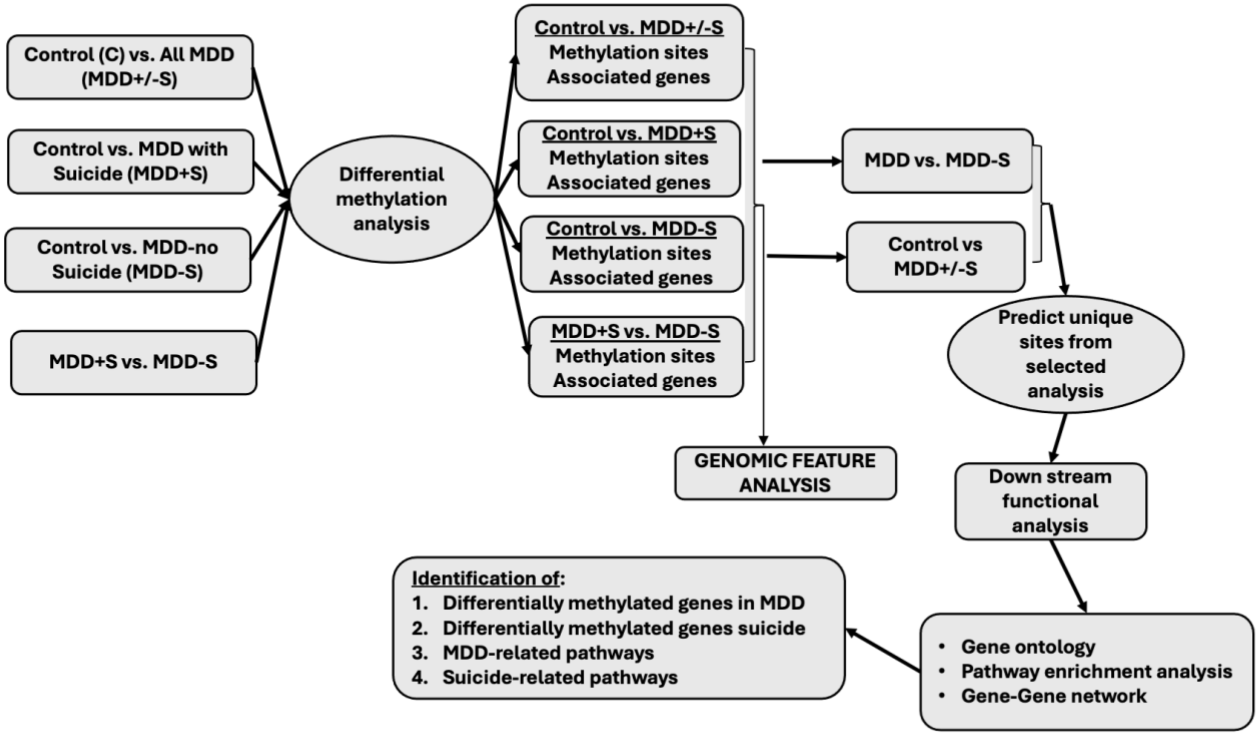

Figure S1: This section provides an overview of the data analysis plan followed in this study. The workflow depicted in the figure outlines the essential steps undertaken to form distinct groups and subsequently analyze them using Illumina DNA microarray (Infinium HumanMethylation450 BeadChip) for genome-wide DNA methylation data. All datasets were examined across four key group comparisons: C vs. MDD+/-S (Control versus all MDD), C vs. MDD+S (Control versus MDD with suicide), C vs. MDD-S (Control versus MDD without suicide), and MDD-S vs. MDD+S (MDD without suicide versus MDD with suicide). Differential methylation changes at specific sites and proximal genes were identified, followed by the mapping of gene ontological functions and pathways, as detailed in the methods section.

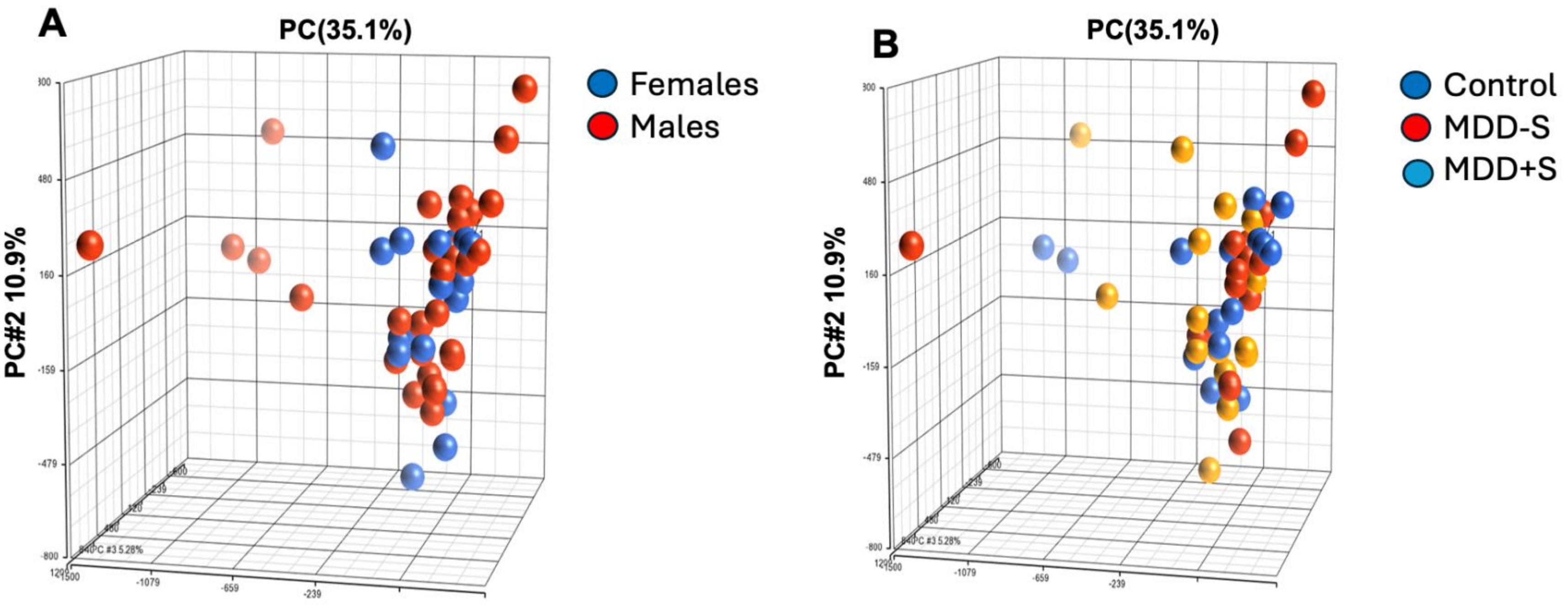

Figure S2: Principal Component Analysis (PCA) plots illustrate the impact of confounding variables on DNA methylation profiles: A) Displaying two discrete clusters representing all sample types, distinguished by gender (Male: Red circle, Female: Blue circle). B) Depicting three discrete clusters corresponding to Control, MDD-S, and MDD+S groups (Control: Blue circle, MDD-S: Red circle, MDD+S: Orange circle).

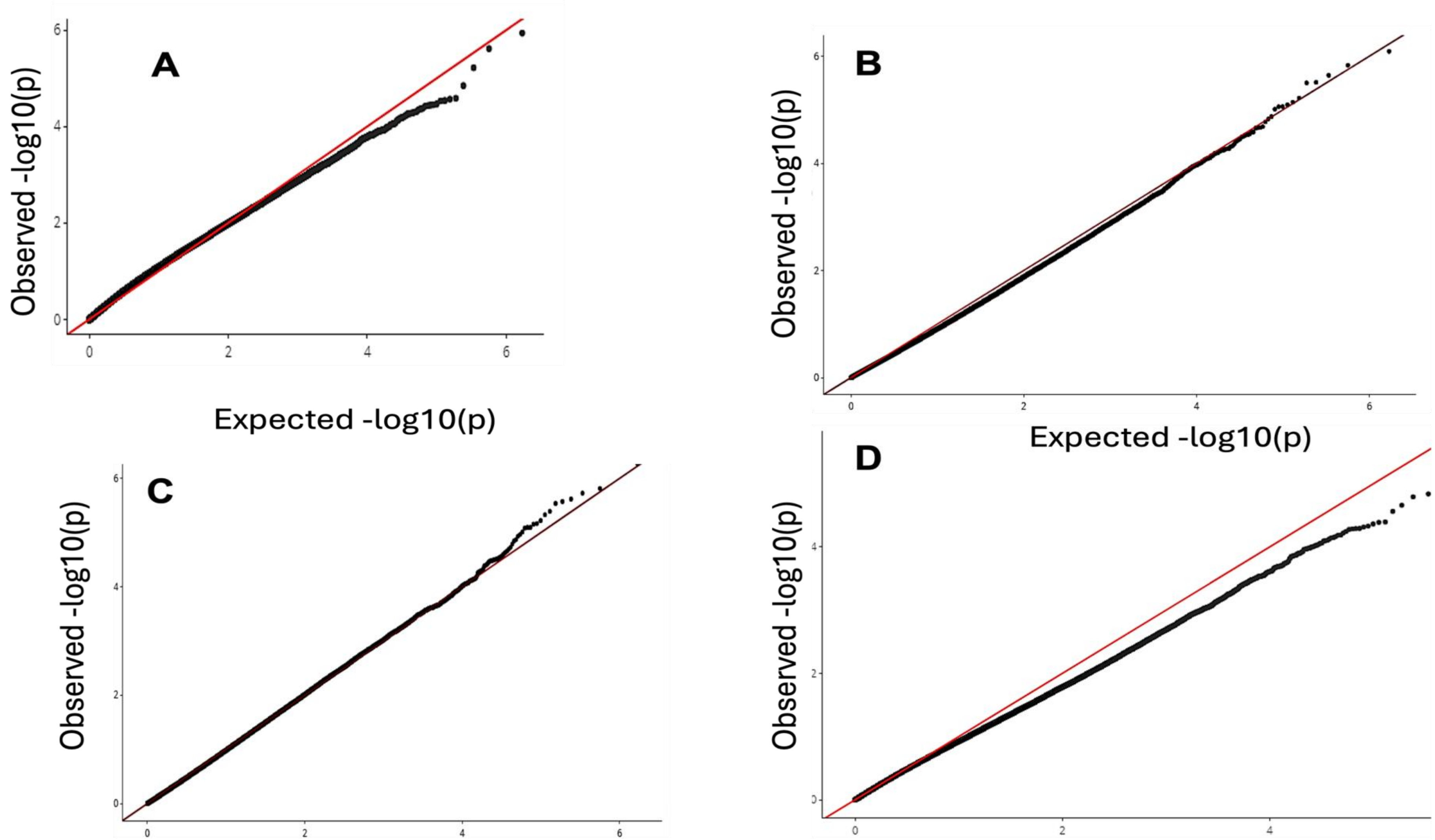

Figure S3: The Q-Q plot illustrates the relationship between observed and expected values across all four groups (C vs. MDD+/-S, MDD-S vs. MDD+S, C vs. MDD-S and C vs. MDD+S) of analysis (A-D). The red line represents the expected values, while the black dots represent the observed values for each methylated site.

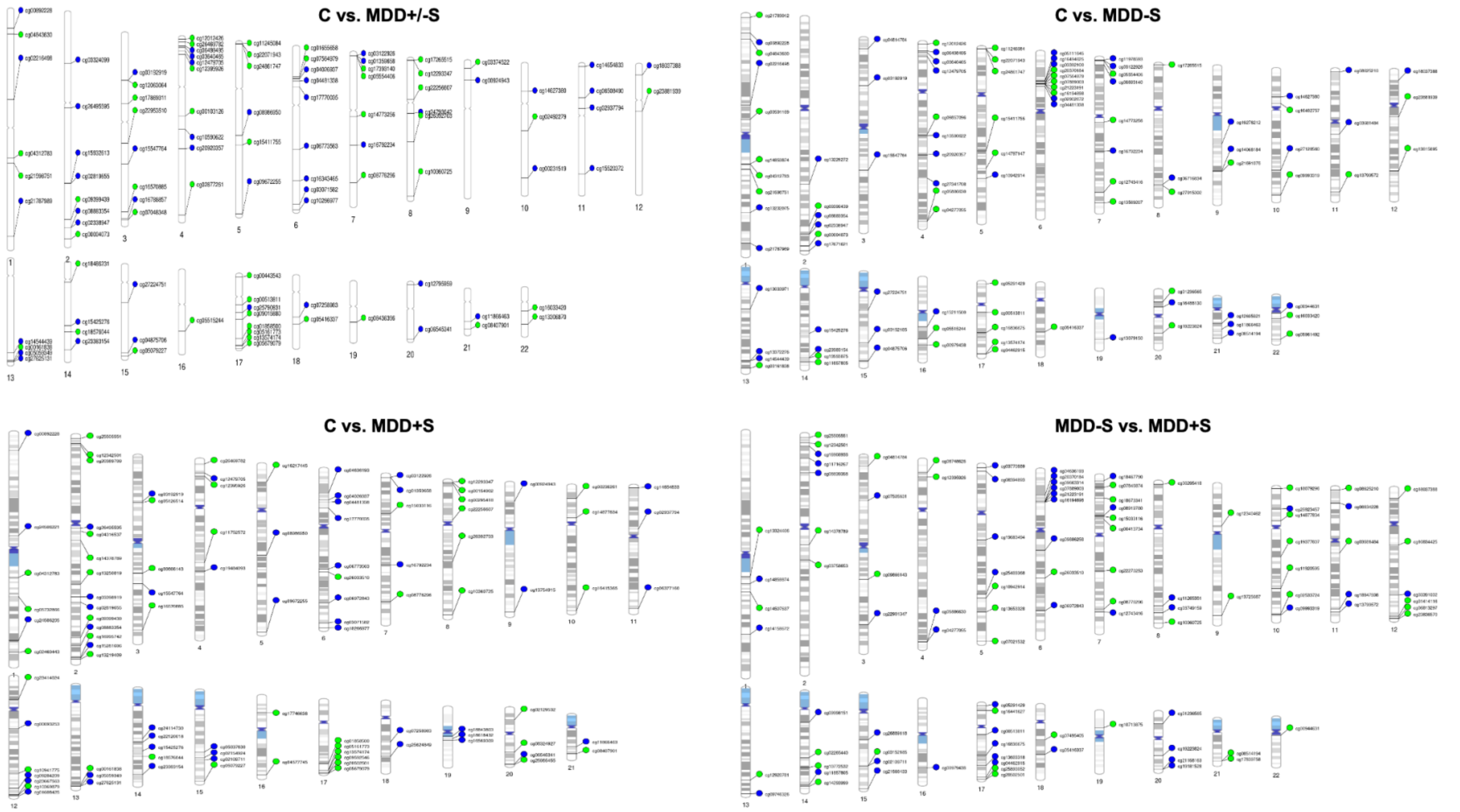

Figure S4: The phenogram displays the chromosome-wise mapping of the top 100 differential methylation sites and genes across all four sets of analyses (C vs. MDD+/-S, MDD-S vs. MDD+S, C vs. MDD-S, and C vs. MDD+S). Significant top 50 differential methylated sites are labeled on individual chromosomes in all four-group analyses. Hypermethylated genes are marked in blue, while hypomethylated genes are marked in green.

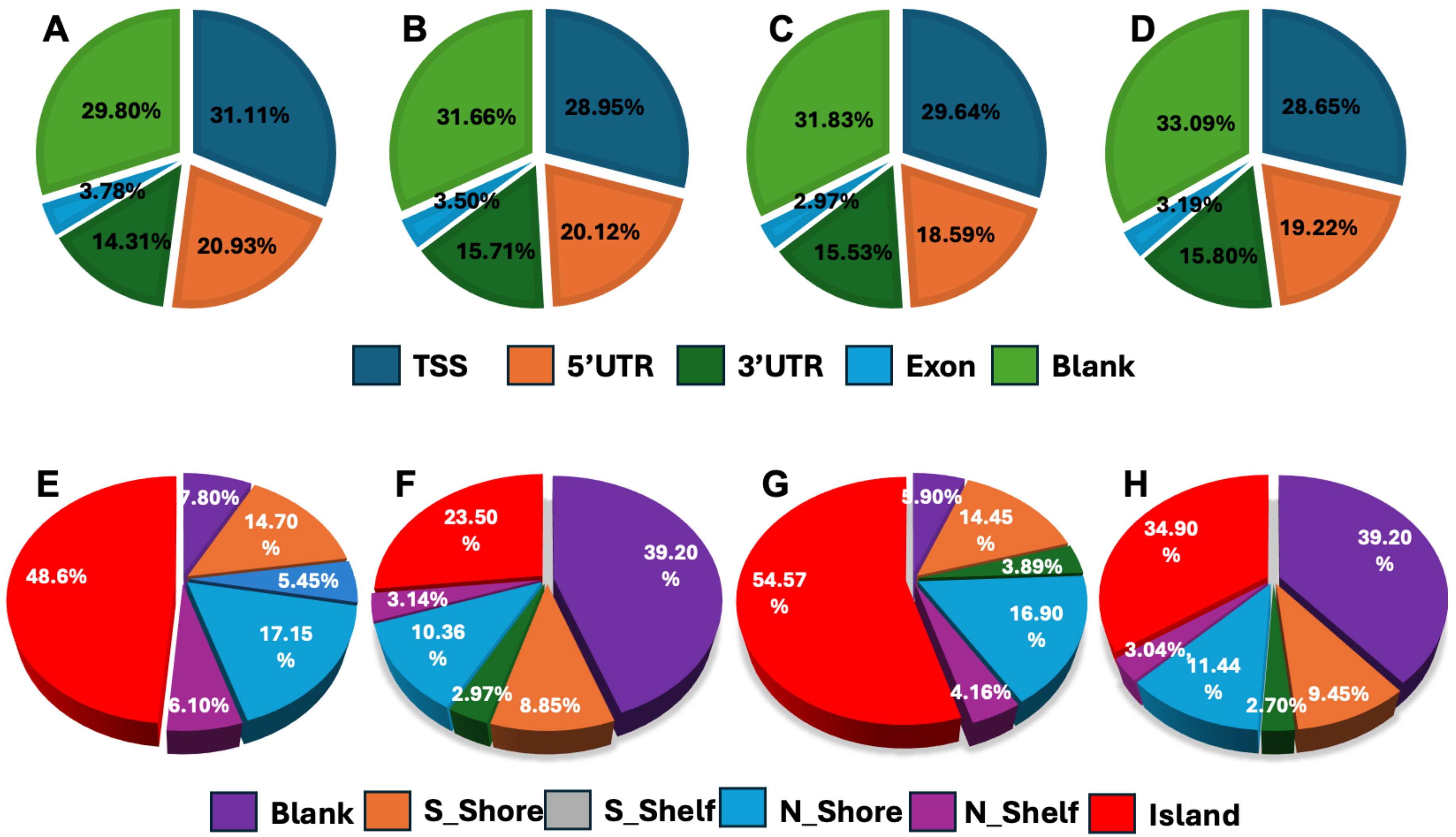

Figure S5: Pie charts in this figure depicts the proportions of genomic features, including TSS, UTR, and exonic regions, associated with differential methylation sites (DMs) identified in the following analyses: A) C vs. MDD+/-S, B) MDD-S vs. MDD+S, C) C vs. MDD-S, and D) C vs. MDD+S. Additionally, panels E-H showcase the genomic distribution of DMs in pairwise comparisons (C vs. MDD+/-S, MDD-S vs. MDD+S, C vs. MDD-S, and C vs. MDD+S), highlighting methylation landmarks (Islands, N_shore, N_Shelf, S_shore, and S_shelf) associated with CpG islands.

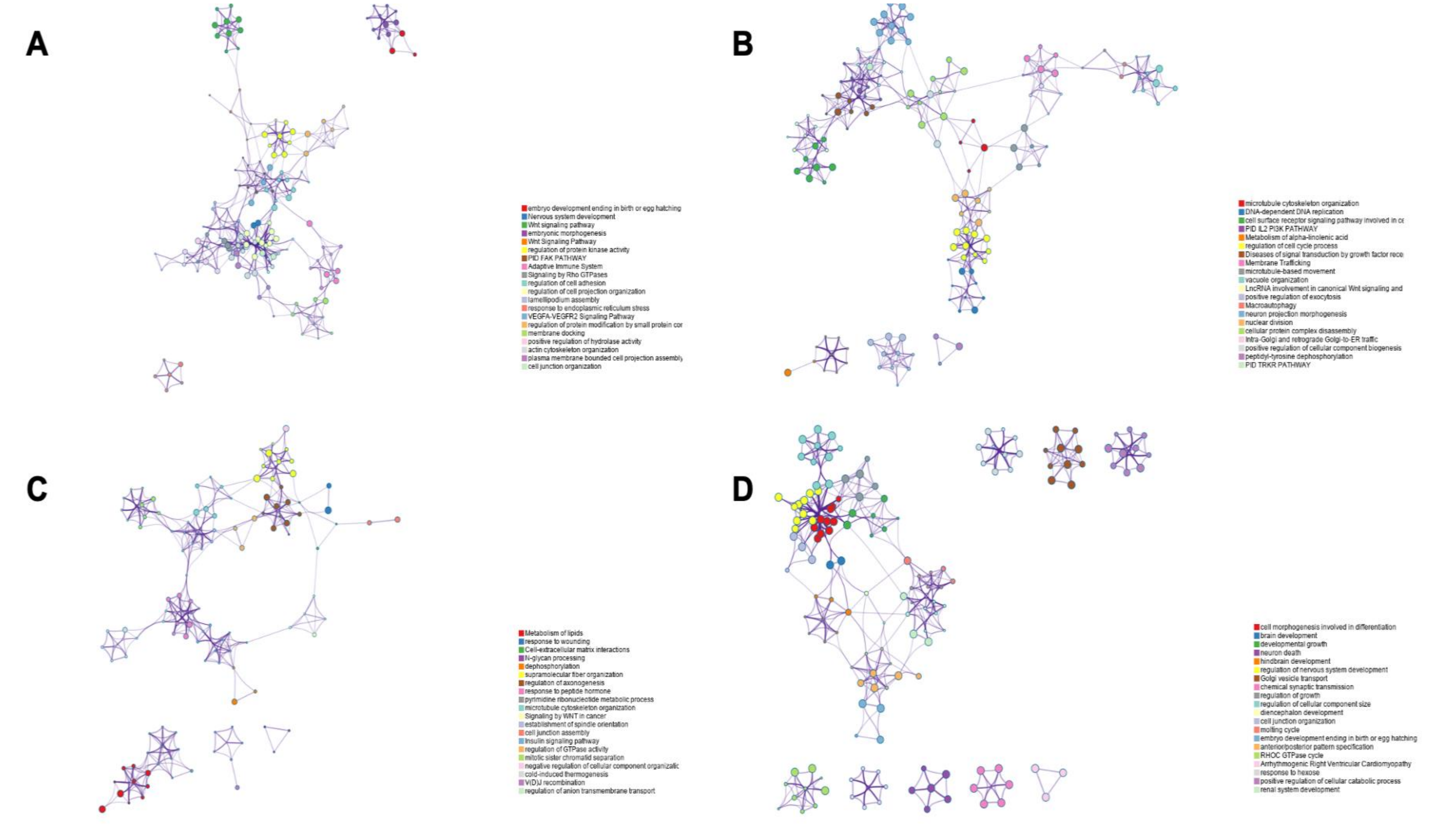

Figure S6: The enrichment network is visualized based on the overrepresented gene list from four sets of analyses (C vs. MDD+/-S, MDD-S vs. MDD+S, C vs. MDD-S, and C vs. MDD+S). Panels A-D depict networks mapped with 50 enriched terms connected by major and minor domains. Each node is color-coded based on its membership in one of the top 50 ontologies, with the size and color intensity of each node corresponding to its statistical significance ($p < 0.05$), where larger and darker nodes indicate greater significance. The inset panels of each central network show the corresponding ontology enrichment list from each analysis.

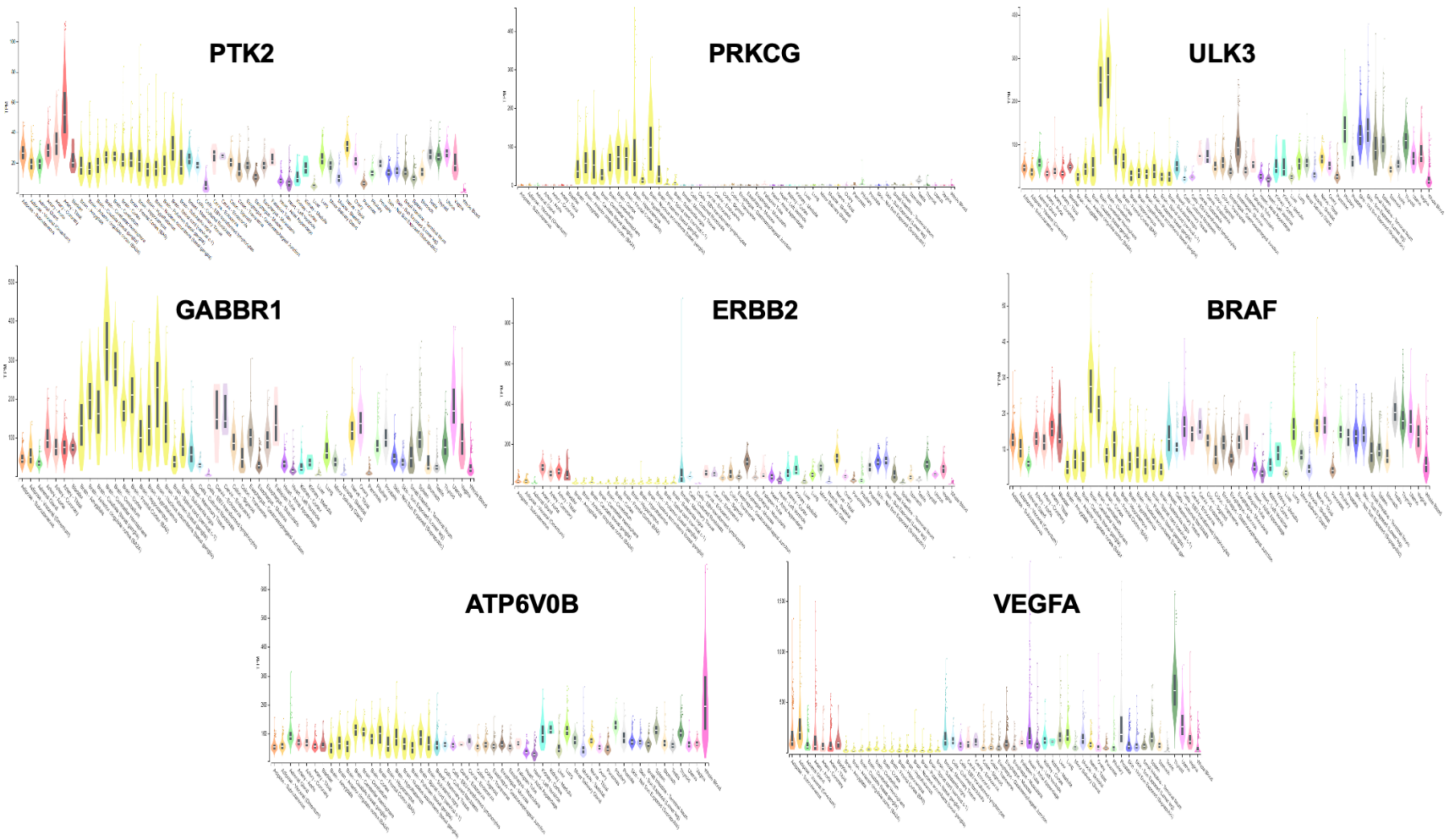

Figure S7: Brain-Specific Expression Enrichment of Key Methylated Genes in the Context of Other Tissues was examined using the Genotype-Tissue Expression (GTEx) database. GTXe aggregates data from tissue-specific gene expression across a spectrum of 54 non-diseased tissue sites from over 1000 individuals. Each key gene identified through GO-based functional enrichment analysis (VEGFA, PTK2, ULK3, PRKCG, ERBB2, GABBR1, ATP6V0B, and BRAF) underwent annotation with GTEx resource data to ascertain its specificity to brain tissues. Violin plots representing all key 8 genes with varying degrees of tissue specificity across distinct brain regions.

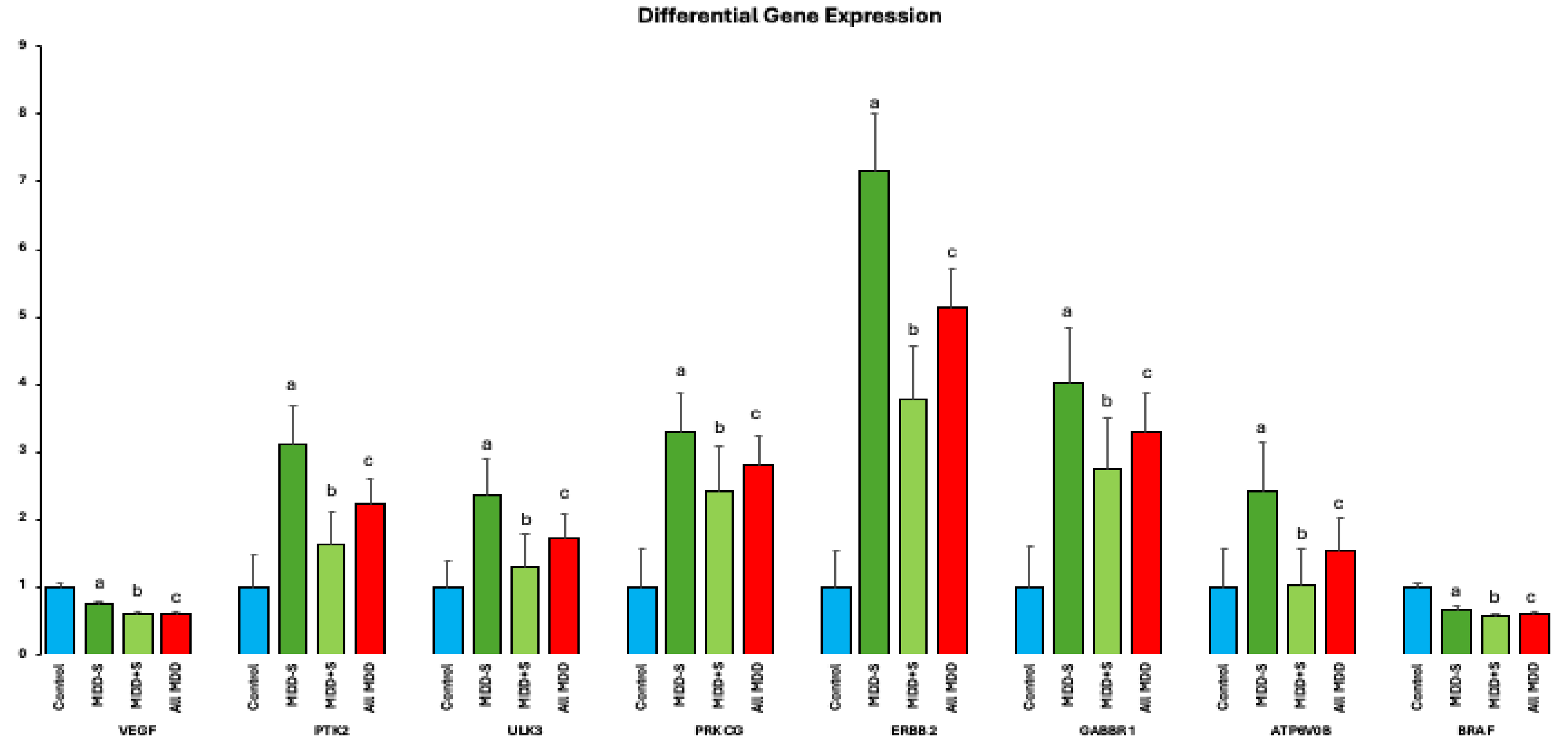

Figure S8: The bar plot displays a significant decrease in expression for VEGFA and BRAF in dlPFC across all three groups (MDD+S, MDD-S, and total MDD) compared to the control group. The other six genes, including PTK2, ULK3, PRKCG, GABBR1, ERBB, and ATP6V0B, showed a significant trend in expression upregulation across all three groups compared to the control group. All data were compared following the ddCT method, and expression fold changes were determined based on GAPDH normalized Ct values. Each bar plot presented the fold change difference ± SEM. The significance level was determined using an independent-sample ‘t’ test (VEGF, a = 0.0373, b = 0.014, c = 0.011; PTK2, a = 0.0364, b = 0.160, c = 0.038; ULK2, a = 0.040, b = 0.279, c = 0.094; PRCK3, a = 0.022, b = 0.079, c = 0.024; ERBB2, a = 0.003, b = 0.026, c = 0.005; GABBR, a = 0.028, b = 0.073, c = 0.029; ATP60B, a = 0.086, b = 0.485, c = 0.203; BRAF, a = 0.018, b = 0.001, c = 0.015).

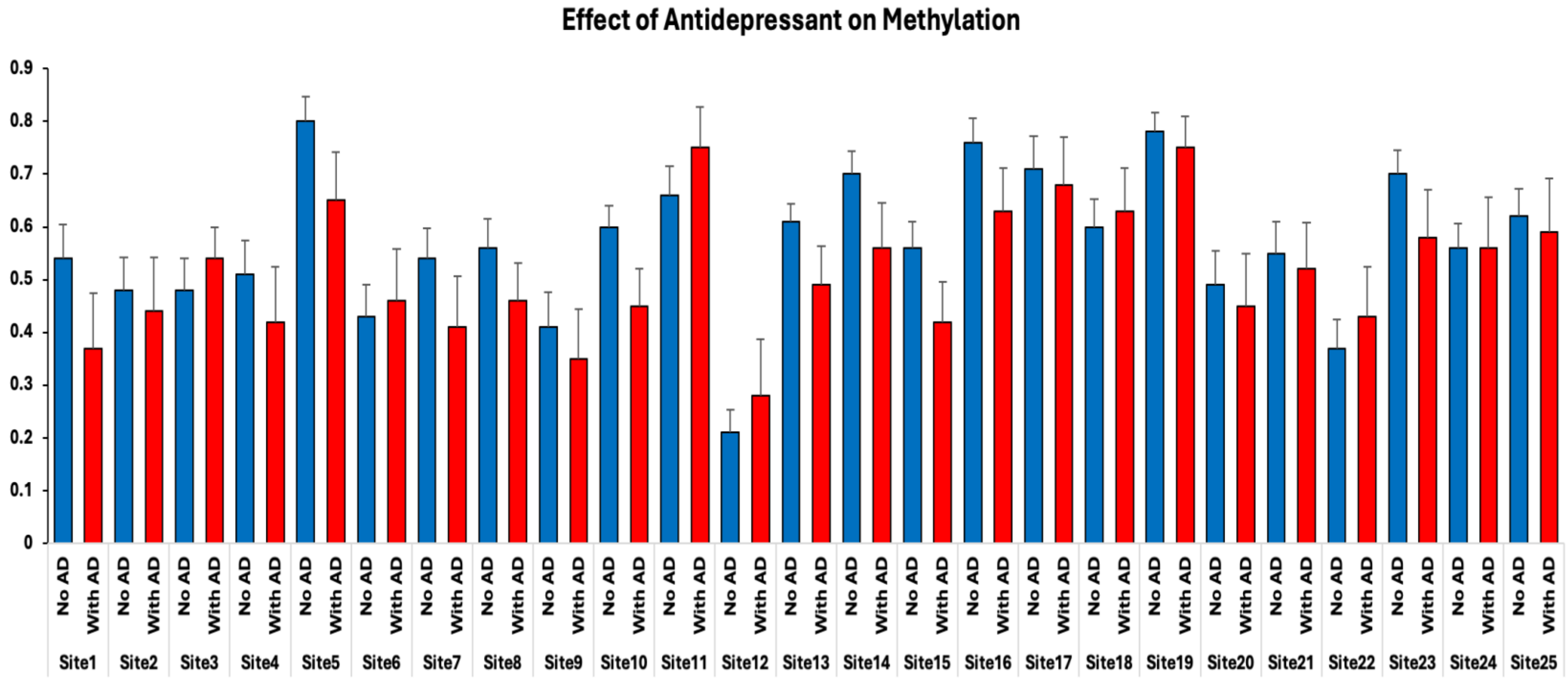

Figure S9: Effect of antidepressants (AD) on top 25 significantly hypermethylated sites in dlPFC of MDD subjects. Each bar plot presents the mean ± SEM. The level of significance was determined using an independent-sample ‘t’ test.

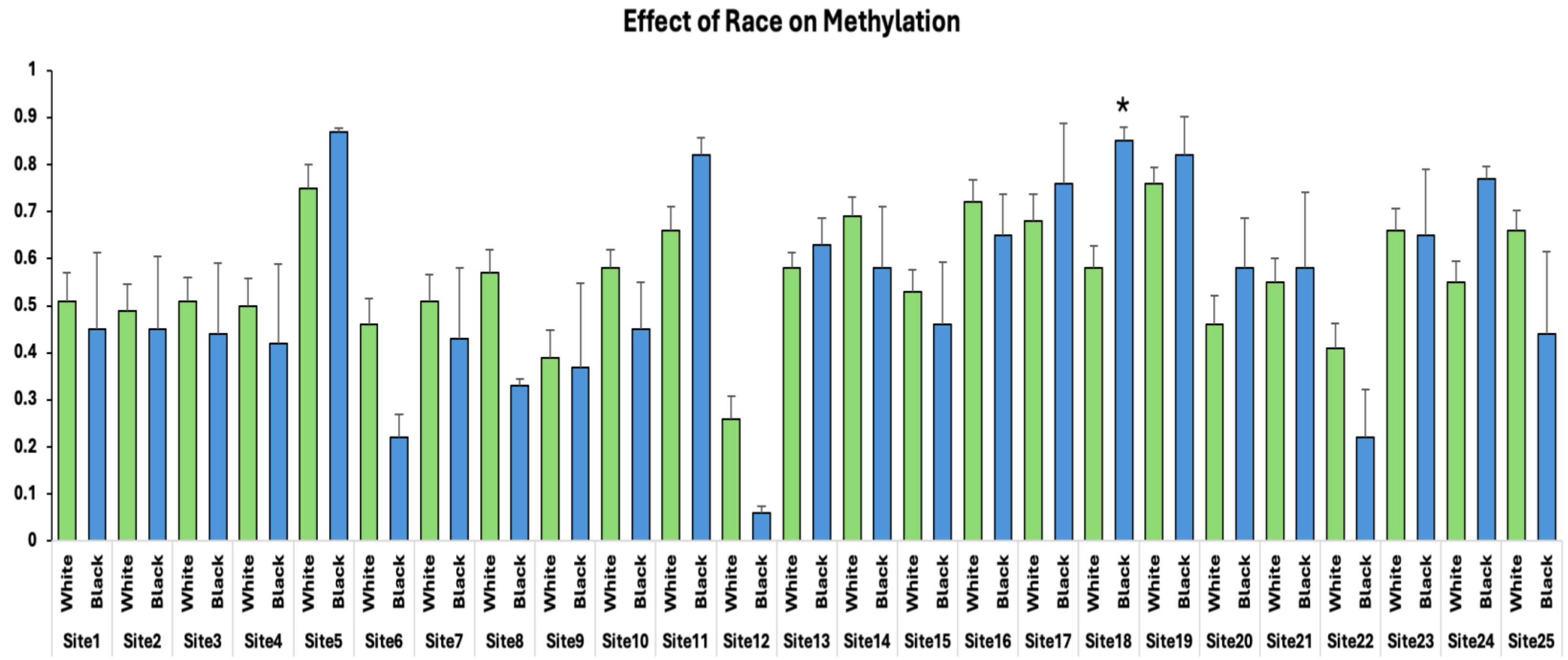

Figure S10: Effect of race on top 25 significantly hypermethylated sites in dlPFC of MDD subjects. Each bar plot presents data in the mean ± SEM. The level of significance was determined using an independent-sample ‘t’ test. ‘*’ denotes a significant difference between MDD and control groups (p<.001).

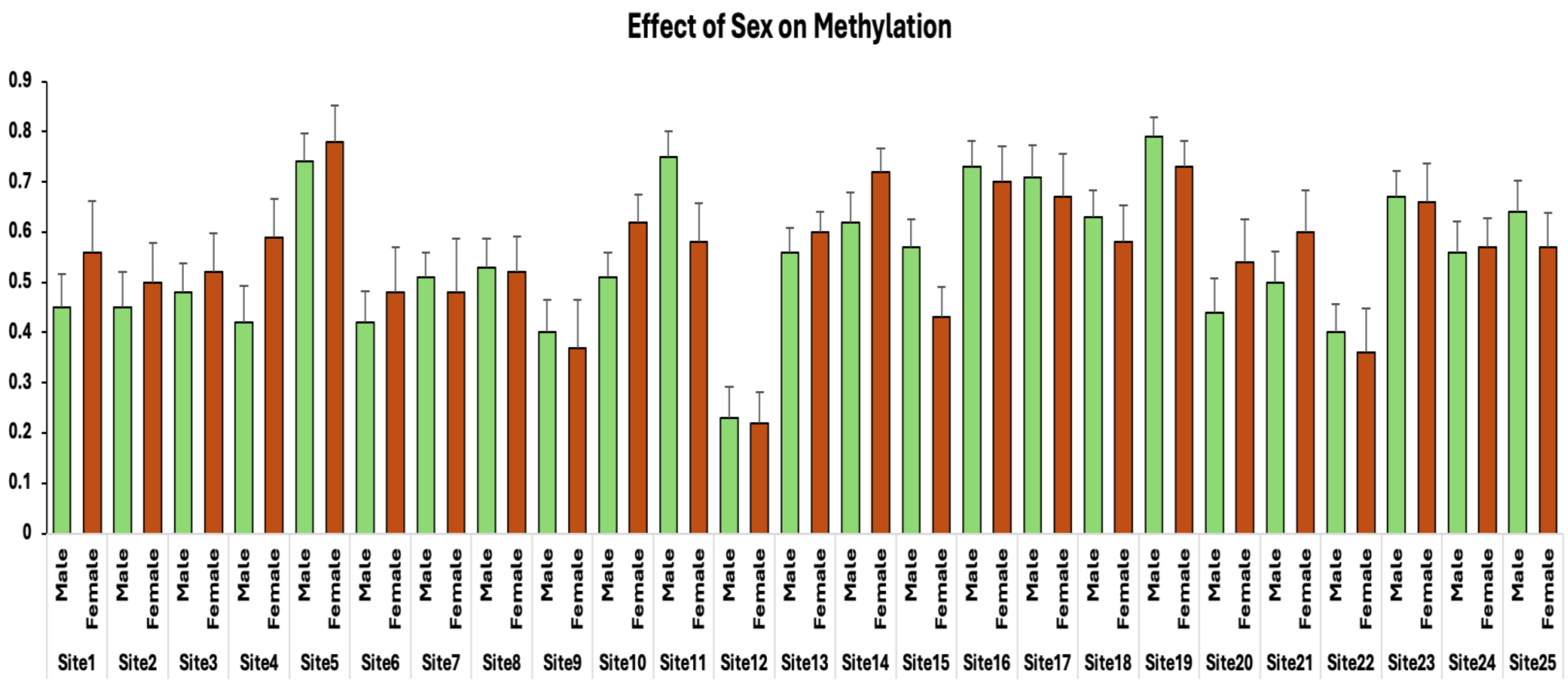

Figure S11: Effect of sex on top 25 significantly hypermethylated sites in dlPFC of MDD subjects. Each bar plot presents data in the mean ± SEM. The level of significance was determined using an independent-sample ‘t’ test.
